# The essential roles of cytidine diphosphate‐diacylglycerol synthase in bloodstream form *Trypanosoma brucei*

**DOI:** 10.1111/mmi.12553

**Published:** 2014-03-28

**Authors:** Alison C. Lilley, Louise Major, Simon Young, Michael J. R. Stark, Terry K. Smith

**Affiliations:** ^1^ Biomedical Sciences Research CentreSchool of BiologyThe University of St. AndrewsThe North HaughSt. AndrewsFife ScotlandKY16 9STUK; ^2^ Centre for Gene Regulation & ExpressionCollege of Life SciencesUniversity of DundeeDundeeScotlandDD1 5EHUK; ^3^ The Novo Nordisk Foundation Center for Protein ResearchUniversity of Copenhagen Faculty of Health and Medical SciencesBlegdamsvej 3Bbuilding 6.1DK‐2200Copenhagen NDenmark

## Abstract

Lipid metabolism in *T**rypanosoma brucei*, the causative agent of African sleeping sickness, differs from its human host in several fundamental ways. This has lead to the validation of a plethora of novel drug targets, giving hope of novel chemical intervention against this neglected disease. Cytidine diphosphate diacylglycerol (CDP‐DAG) is a central lipid intermediate for several pathways in both prokaryotes and eukaryotes, being produced by CDP‐DAG synthase (CDS). However, nothing is known about the single *T**. brucei* CDS gene (Tb927.7.220/EC 2.7.7.41) or its activity. In this study we show TbCDS is functional by complementation of a non‐viable yeast CDS null strain and that it is essential in the bloodstream form of the parasite via a conditional knockout. The TbCDS conditional knockout showed morphological changes including a cell‐cycle arrest due in part to kinetoplast segregation defects. Biochemical phenotyping of TbCDS conditional knockout showed drastically altered lipid metabolism where reducing levels of phosphatidylinositol detrimentally impacted on glycoylphosphatidylinositol biosynthesis. These studies also suggest that phosphatidylglycerol synthesized via the phosphatidylglycerol‐phosphate synthase is not synthesized from CDP‐DAG, as was previously thought. TbCDS was shown to localized the ER and Golgi, probably to provide CDP‐DAG for the phosphatidylinositol synthases.

## Introduction

**Figure 1:**
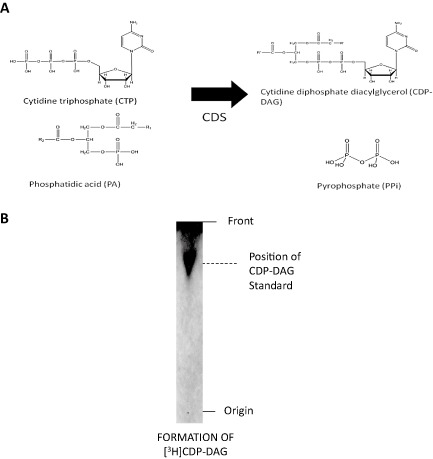
CDP‐DAG synthase activity. A. Schematic of the enzymatic reaction catalysed by CDP‐DAG synthase. B. TbCDS assay of *T. brucei* lysate, autoradiography of a representative HPTLC of lipid fraction shows [^3^H]CDP‐DAG formation from [^3^H]CTP and PA. The position of unlabelled CDP‐DAG standard run in the same solvent system and visualized by iodine is indicated on the right hand side.

*Trypanosoma brucei*, the causative agent of African sleeping sickness, expresses a variant surface glycoprotein (VSG) coat, which protects the parasite from the alternative complement pathway and from a specific immune response by antigenic variation (Cross, 1996). The 5 × 10^6^ VSG dimers are held in the outer leaflet plasma membrane of the parasite by glycosylphosphatidylinositol (GPI) anchors. The biosynthesis of this conserved GPI anchor has been previously genetically (Nagamune *et al*., 2000; Chang *et al*., 2002) and chemically validated (Smith *et al*., 2004) as a drug target in bloodstream form *T. brucei.* The importance of GPI anchors to *T. brucei* has lead our group to investigate various aspects of lipid metabolism, some enzymes of which have already been shown to be essential (reviewed in Smith and Buetikofer, 2010). The nucleolipid cytidine diphosphate diacylglycerol (CDP‐DAG) is a key metabolite, acting as a high energy donor in the synthesis of numerous phospholipids in both prokaryotes and eukaryotes. In *T. brucei* these include synthesis of phosphatidylinositol (PI), which is essential for the production of GPI anchors (Martin and Smith, 2006b), and potentially phosphatidyglycerol (PG), cardolipin (CL) and phosphatidylserine (PS). CDP‐DAG is synthesized by the enzyme CDP‐DAG synthase (CDS) (EC 2.7.7.41) from cytidine triphosphate (CTP) and phosphatidic acid (PA) (Fig. 1A).

A gene encoding CDS activity was first discovered in *E. coli* mutants deficient in CDP‐DAG synthesis (Ganong *et al*., 1980; Ganong and Raetz, 1982). In *Drosophila* a photoreceptor CDS is involved in phosphoinositide signalling and vision (Wu *et al*., 1995), which increased interest in the gene and led to its cloning and study in yeast (Shen *et al*., 1996) and a variety of mammals (Heacock *et al*., 1996; Saito *et al*., 1997; Halford *et al*., 1998; Volta *et al*., 1999; Inglis‐Broadgate *et al*., 2005). CDS homologues have now been identified in all eukaryotic genomes sequenced to date and some organisms have multiple CDS genes (Lykidis, 2007). The genes show high sequence similarity, suggesting a degree of conservation during evolution. All identified CDS enzymes are integral membrane proteins. In prokaryotes, CDS activity is associated with the inner membrane of the cell envelope (Bell *et al*., 1971; White *et al*., 1971) and in eukaryotes the majority of activity has been found in the ER and mitochondria (Bishop and Strickland, 1976; Kuchler *et al*., 1986; Kelley and Carman, 1987; Mok *et al*., 1992; Heacock and Agranoff, 1997).

The fact that the pool of CDP‐DAG is much smaller than that of CDS's lipid substrate PA (Raetz and Kennedy, 1973; Thompson and MacDonald, 1975; Nickels *et al*., 1994) along with the position of CDS at a branch point in phospholipid synthesis suggests CDP‐DAG synthesis may be a rate‐limiting step. In yeast, CDS is under tight transcriptional regulation via the inositol‐responsive *cis*‐acting element and *trans*‐acting factors (reviewed in Carman and Han, 2011), but in other organisms regulation varies greatly (Carter and Kennedy, 1966; Sribney *et al*., 1977; Langley and Kennedy, 1978; Ganong and Raetz, 1982; Baelee and Carman, 1984; Icho *et al*., 1985; Kelley and Carman, 1987; Carman and Baelee, 1992; Mok *et al*., 1992; Shen and Dowhan, 1996; Lykidis *et al*., 1997; Saito *et al*., 1997; Sato *et al*., 2000; Haselier *et al*., 2010).

CDS null mutants in yeast (Shen and Dowhan, 1996; Shen *et al*., 1996), *E. coli* (Ganong and Raetz, 1982) and *Synechocystis* (Sato *et al*., 2000) are inviable due to massive direct and indirect disruption of phospholipid synthesis. Due to the essential requirement for PI (Martin and Smith, 2006a,b), phosphorylated PIs (PIPs) (Rodgers *et al*., 2007) and GPI anchors (Nagamune *et al*., 2000; Chang *et al*., 2002; Smith *et al*., 2004) in *T. brucei*, it seemed likely that CDS would be essential for the parasite, therefore presenting a possible novel drug target. In order to validate CDS as a drug target we created a conditional knockout in the bloodstream form parasite, which has highlighted several novel aspects of phospholipid metabolism in this early divergent eukaryotic parasite.

## Results

### Cloning and sequencing *T**. brucei* CDS (TbCDS)

A putative CDS was identified in the *T. brucei* genome database (Sanger Centre) using Blast (Tb927.2.220). This putative ORF was PCR amplified, cloned and sequenced. The sequence and its translation are shown in Fig. S1. An alignment of the predicted translated sequence with CDSs from other organisms is shown in Fig. S2A, with a corresponding phylogenetic tree in Fig. S2B. Unsurprisingly, TbCDS is closely related to other kinetoplastids, showing 99% identity with the *T. b. brucei* strain 927 and *T. b. gambiense*; 64% with *T. cruzi*, 60% with *T. vivax* and 49–50% with various *Leishmania* species, while significantly less to the two human CDS homologues. The size of the predicted TbCDS protein is 46 kDa, which is in good agreement with other eukaryotic CDSs such as *Arabidopsis thaliana* CDS5 at 39.9 kDa (Haselier *et al*., 2010) and the human CDS1 at 46.1 kDa (Heacock *et al*., 1996).

TbCDS is highly hydrophobic with a grand average of hydropathicity of 0.285 (Kyte and Doolittle, 1982) with 7 predicted transmembrane domains (highlighted in Fig. S1). This is consistent with all identified CDS enzymes which are integral membrane proteins with multiple membrane spanning domains (Carman and Kelley, 1992; Heacock *et al*., 1996; Saito *et al*., 1997; Volta *et al*., 1999; Martin *et al*., 2000; Nigou and Besra, 2002). The TbCDS protein contains the typical phosphatidate cytidyltransferase signature: S‐x‐[LIVMF]‐K‐R‐x(4)‐K‐D‐[GSA]‐x(2)‐[LIF]‐[PGS]‐x‐H‐G‐G‐[LIVMF]‐x‐D‐R‐[LIVMFT]‐D (highlighted yellow in Fig. S1A), which is shared by almost all predicted homologues, both eukaryotic and prokaryotic (Koonin, 1996; Ponting and Kerr, 1996).

BLAST searches using typical prokaryotic CDS sequences recovered a putative second CDS gene in *Trypanosoma cruzi* (Tc00.1047053508707.140 /TcCLB.508707.140), *Trypanosoma vivax* (TvY486_0025800) and several *Leishmania* species (LinJ.32.3010, LmjF.32.2870, LmxM.31.2870, LtaP32.3050) but not in *T. brucei*. All of these genes are more similar to the prokaryotic than the eukaryotic CDS genes. An alignment of these predicted proteins with some typical prokaryotic CDS sequences are shown in Fig. S3A with a corresponding phylogenetic tree in Fig. S3B. This shows that the protozoan homologous have N‐terminal extensions as well as several insertions, making these gene products significantly larger than the average prokaryotic CDS (Agabian, 1990).

It is assumed that organisms that have multiple copies of CDS, i.e. humans and *Arabidopsis*, target them to different cellular locations, providing local concentrated pools of CDP‐DAG to be used by different phospholipid pathways. This may also be true for *T. cruzi* and *Leishmania* that have both a eukaryotic and prokaryotic version, but *T. brucei* have likely lost their prokaryotic homologue in the recent past.

### Rescue of yeast of CDS null

**Figure 2:**
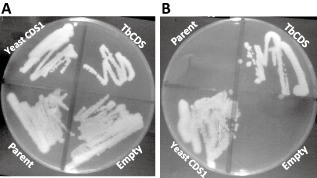
Plasmid shuffle shows TbCDS can complement the yeast cds1::KanMX knockout. A. After transformation of haploid *cds1::KanMX* [pRS425‐MET25*‐CDS1*] with empty p405‐TEF1, p405‐TEF1‐*TbCDS* or p405‐TEF1‐*CDS1* and selection for both plasmids, the three strains plus the *cds1::KanMX* [pRS425‐MET25*‐CDS1*] parent strain were plated on YPD, where all strains grew normally. B. Strains were then plated onto YPD +5‐FOA in order to select against the pRS425‐MET25*‐CDS1* plasmid. Parent = *cds1::KanMX* [pRS425‐MET25‐*CDS1*]; Empty = Parent transformed with p405‐TEF1; Yeast = Parent transformed with p405‐TEF1‐*CDS1*; TbCDS = parent transformed with p405‐TEF1‐TbCDS.

To confirm that the *T. brucei* CDS homologue encoded for a functional CDS, it was used to rescue viability of an otherwise inviable yeast *cds1* null strain. A schematic of the strategy used is shown in Fig. S4. Briefly, a diploid yeast strain heterozygous for a *cds1* gene knockout (*CDS1/cds1::KanMX*) containing yeast *CDS1* on the *URA3*‐marked pRS426‐MET25 plasmid was used to generate haploid *cds1* knockout strains that were complemented by the plasmid. As yeast *CDS1* is an essential gene, loss of this plasmid resulted in lethality so these strains could not grow in the presence of 5‐fluoro‐orotic acid (5‐FOA), which selects against the *URA3* marker on the plasmid (Fig. S5 – compare A and B). Using a second, *LEU2*‐marked plasmid (p405‐TEF1), either *TbCDS* or yeast *CDS1* were introduced into this background. These strains, along with the parent strain and transformants carrying the empty p405‐TEF1 vector, were tested for growth after counterselection for pRS426‐MET25‐*CDS1* on 5‐FOA. Only the strains containing either yeast *CDS1* or TbCDS on the *LEU2* selectable plasmid were viable, while the parent strain with and without the empty plasmid was non‐viable (compare Fig. 2A and B). Lipidomic analysis by electrospray mass spectrometry of the yeast parent strain and the *CDS1* null expressing either the ectopic yeast *CDS1* or TbCDS on a plasmid showed alterations in their phospholipid profiles. This may reflect a different acyl preference on the substrate PA (Fig. S6A–C) resulting in slightly different CDP‐DAG species being formed and thus variation in the relative ratio of the PI species formed (compare species at 808 m/z and 836 m/z), which may impact on inositolphosphoceramide (IPC) formation and thus lipid homeostasis in the resulting yeast strains (Carman and Fischl, 1992). Collectively, this functional complementation provides definitive evidence that TbCDS encodes a functional CDS enzyme as it is able to rescue an otherwise non‐viable yeast *cds1* null mutant.

### Is CDS essential in bloodstream form *T**. brucei*?

According to the TriTrypDB, the TbCDS (Tb427.07.220) is expressed equally throughout the various life cycles stages, and this was confirmed in strain 427 by RT‐PCR in both the bloodstream and procyclic forms (Fig. S7). In addition, washed bloodstream *T. brucei* membranes exhibited CDS activity, as they were able to catalyse the conversion of [^3^H]CTP and PA into [^3^H]CDP‐DAG product (Fig. 1B).

To ascertain if TbCDS is an essential gene in *T. brucei*, a conditional TbCDS knockout was created. As the ‘wild type’ cell line used here constitutively expresses the T7 RNA polymerase and the tetracycline repressor protein, a tetracycline inducible (Ti) haemagglutinin (HA)‐tagged ectopic copy of the TbCDS was introduced (pLew 100) into the cell line prior to deletion of both TbCDS alleles in the presence of tetracycline, allowing the creation of a conditional double knockout (Wirtz *et al*., 1999). The resulting *TbCDS*‐*HA*^Ti^ Δ*TbCDS::PAC/*Δ*TbCDS::HYG* clone was obtained and the genotype of the cell‐line confirmed by PCR (Fig. S8A), while expression of the ectopic TbCDS copy was confirmed as being tetracycline inducible by anti‐HA Western blot (Fig. S8B). Please note, the apparent mobility of CDS anti‐HA band is significantly higher than the predicted ∼ 47 kDa, as is commonly seen for multiple transmembrane domain proteins on SDS‐PAGE.

**Figure 3:**
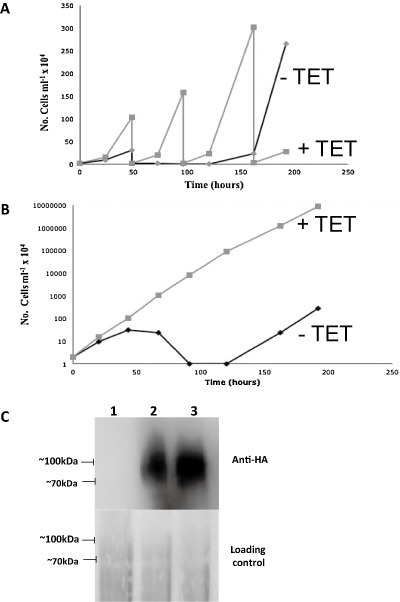
Growth curves and Western blot of the TbCDS conditional double knockout cell line. Cells were washed with tetracycline‐free HMI‐9, transferred to tetracycline‐free media and counted daily. Growth curves are shown for wild type cells in this media. A. Growth curves of *T. brucei* CKO in the absence (black/−TET) or presence (grey/+TET) of tetracycline (A) actual number of cells per ml of culture × 10^4^ after number of hours cultured in the absence (black/−TET) or presence (grey/+TET) of tetracycline. B. Growth curves of log cumulative number of cells over time in the presence and absence of tetracycline. These growth curves were done numerous times and were highly reproducible. C. Anti‐HA western of *T. brucei* CKO either after 48 h without tetracycline (lane 1), or with tetracycline (lane 2), or 150 h without tetracycline (lane 3). Reoccurrence of HA‐tagged TbCDS despite the absence of tetracycline suggests regrowth is due to cells reverting and breaking the tetracycline control of TbCDS expression. Loading control is ponceau stained Western blotted membrane.

To establish whether *CDS* is an essential gene, the growth of the *TbCDS‐HA^Ti^* Δ*TbCDS::PAC/*Δ*TbCDS::HYG* (TbCDS CKO) cell line was monitored in tetracycline‐free HMI‐9 in the absence or presence of tetracycline (Fig. 3A and B). In the absence of tetracycline a growth defect was apparent after 24 h, and after 48 h cells started to die and no visible TbCDS‐HA protein was observed (Fig. 3C, lane 1). By 100 h the cell numbers were below the limits of detection by light microscopy, however, some live cells were visible after 120 h, following which growth resumed as normal. The growth pattern observed was highly reproducible. This revertant growth was likely due to a loss of tetracycline control, as has previously been shown in knockouts of essential genes (Chang *et al*., 2002; Martin and Smith, 2006a), and was confirmed by anti‐HA Western blot showing production of the HA‐tagged TbCDS in the absence of tetracycline (Fig. 3C, lane 3).

### Morphological phenotype of the CDS conditional knockout

**Figure 4:**
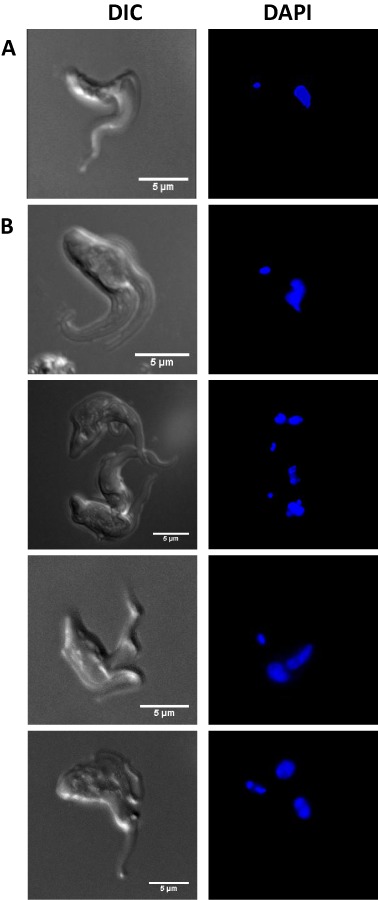
Comparison of wild‐type and TbCDS CKO cell morphology. Projections of Z‐series images taken by light microscopy. Left panel shows DIC and right panel shows DAPI staining and. Cells were fixed with 4% paraformaldehyde, allowed to adhere to poly‐lysine coated slides and mounted with anti‐fade GOLD reagent containing DAPI. A. Wild‐type cells harvested from mid log culture and fixed in 4% paraformaldehyde. B. TbCDS CKO cells harvested from culture 48 h after the removal of tetracycline.

There were morphological changes in the TbCDS CKO cell line after 42 h in the absence of tetracycline compared with wild type cells (Fig. 4). In many of the trypanosomes there was an apparent cell cycle arrest after replication of the flagellum and the formation of a furrow. A pre‐cytokinesis stall is not uncommon in *T. brucei* mutants, but the stall usually occurs before furrow formation (Hammarton *et al*., 2007). DAPI staining revealed that in most cases these cells had stalled at 1K2N, often with enlarged kinetoplast and nuclei. This 1K2N phenotype is surprising given the kinetoplast replicates prior to the nucleus and suggests a defect in kinetoplastid segregation. This downstream affect of depleted CDP‐DAG levels suggests key structural and/or signalling phospholipids may play an important part in correct kinteoplastid DNA replication/segregation. This could be at the tripartite attachment zone, where the basal body is physically attached to the kinetoplast, passing through both the cell and mitochondrial membranes. A very similar, but not identical phenotype was observed in the knock‐down of *T. brucei* MOB1, which caused a stall in cytokinesis through an inhibitory effect on kinetoplast segregation (Hammarton *et al*., 2005).

### Biochemical phenotype of the TbCDS conditional knockout

**Figure 5:**
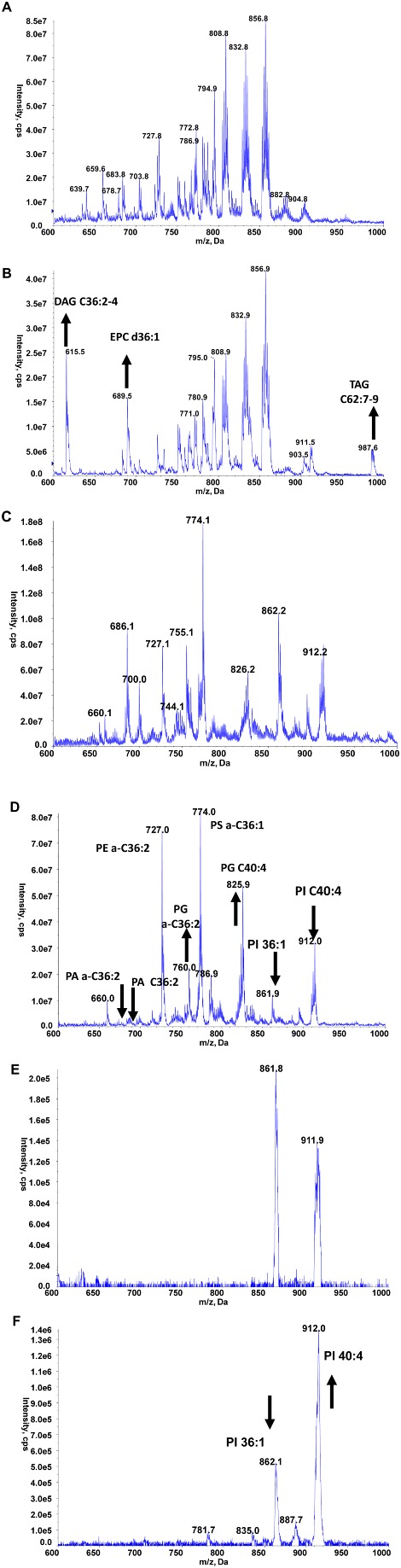
Lipidomic analyses by electrospray mass spectrometry. A and B. Mass spectrometric analyses: Positive ion survey of total phospholipids from wild‐type (A) and TbCDS CKO in absence of tetracycline for 42 h (B). C–F. Negative ion survey of total phospholipids from wild‐type (C) and TbCDS CKO cells in absence of tetracycline for 42 h (D). Parent‐ion scanning of the collision induced fragment at 241 m/z of wild‐type single marker cells (E) and TbCDS CKO cells in absence of tetracycline for 42 h (F). The data presented here are representative scans from one set of samples, but these analyses were carried out on several different samples along with their corresponding controls.

Total lipids were extracted from TbCDS CKO cells grown in the absence (42 h) or presence of tetracycline and analysed by electrospray‐mass spectrometry. As expected, lipid extracts from TbCDS CKO grown in the presence of tetracycline have a similar distribution of molecular species to that of wild‐type cells across the phospholipid classes, PI, PA, PG, PS, PE, PC and sphingolipids to that of wild‐type cells (data not shown). Comparison of the positive ion mode survey scans of TbCDS CKO in the presence and absence of tetracycline shows the relative proportion of the PC and SM remain relatively unchanged (Fig. 5A and B). Some notable differences were an increase in DAG 36:2–4 (615.5 m/z) and an increase in TAG C62:7–9 (987.6 m/z) (Fig. 5B). The negative ion mode survey scans of TbCDS CKO in the presence and absence of tetracycline show dominant ions at 727.1 and 774.1 m/z (Fig. 5C and D) corresponding to the major plasminogen PE (a‐36:1) and PS (a‐36:1) respectively. In the absence of tetracycline two species at 686.1 and 700.0 m/z identified by fragmentation as PA a‐C18:0/C18:2 (Fig. S9A), and its diacyl counterpart PA C18:0/C18:2 (Fig. S9B) are both absent compared with cells grown the presence of tetracycline. These reductions coincide with a significant increase in several PG species including those at 760.0 and 825.9 m/z identified as including a‐C18:0/C18:2 and C18:0/C22:4 respectively (Fig. S9C and D).

Finally, the absence of tetracycline gives rise to a significant decrease in the major PI species at 861 and 912 m/z in corresponding to C36:1 and C40:4 respectively (Fig. 5C and D), with a disproportional reduction between these major PI species. To investigate this further, parent‐ion scanning of m/z 241 (collision‐induced fragmentation, forming inositol‐1,2‐cyclic phosphate) in negative ion mode which only shows the PI species (Fig. 5E and F) allowed the relative ratio to be calculated, showing a decrease from 1.29 to 0.38 (861 to 912 m/z). Quantification of the total *myo*‐inositol containing phospholipids by GC‐MS showed that in the absence of tetracycline there was only 57 ± 6% *myo*‐inositol containing lipids compared with cells grown in the presence of tetracycline.

The decrease in CDS activity obviously causes a marked reduction in PI levels, however the disproportional reduction implies the C36:1 species turns over faster than the C40:4 species. No significant disproportional reduction was observed in our previous work on either INO1 (Martin and Smith, 2006a) or PI synthase (Martin and Smith, 2006b). This study may indicate that that the cellular location of either the synthesis or downstream use of the PI species may be different. Alternatively, it may highlight an issue with availability of certain CDP‐DAG species under conditions of limiting CDS activity.

The biochemical phenotype of the TbCDS CKO cells was also investigated by *in vivo* radiolabelling experiments with [^3^H]myristate, [^3^H]glycerol and [^3^H]inositol after the cells had been grown either in the presence or absence of tetracycline for 42 h and compared with wild‐type cells. After labelling, lipids were extracted, desalted, separated by high performance thin layer chromatography (HPTLC), and detected by fluorography.

Labelling of the *TbCDS* CKO cells grown in the absence of tetracycline with [^3^H]myristate (Fig. S10A) showed no significant decrease to those grown in the presence of tetracycline, although there was a slight increase in the labelling of neutral lipids (diacylglycerol and triacylglycerol), confirming the observed increase in the positive ion mode survey scan (Fig. 5B). This is also a clear indication that at this point under permissive conditions the cells are still metabolically active. The incorporation of [^3^H]glycerol into different glycerolipids (Fig. S10B) was considerably altered in the TbCDS CKO cells in the absence of tetracycline. There is significantly less labelling of PE and PI in the knockout, but PC seems unaffected, while PG seems to have increased. Surprisingly, the incorporation of [^3^H]inositol into PI and PIP seems relatively unaffected in the TbCDS CKO (Fig. S10C), despite the [^3^H]glycerol labelling showing a decrease in newly formed PI. However, this anomaly is likely due to head‐group swapping with pre‐existing unlabelled PI by the PI synthase as observed previously (Martin and Smith, 2006a).

**Figure 6:**
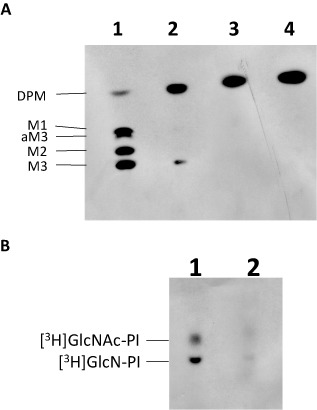
TbCDS is essential for formation of GPI intermediates. A. Cell free system of wild‐type (lanes 1 and 3) and TbCDS CKO cells in absence of tetracycline for 42 h (lanes 2 and 4), membranes were incubated with GDP‐[^3^H]Man in the presence (lanes 1 and 2) and absence (lanes 3 and 4) of UDP‐GlcNAc. B. Cell free system of wild‐type (lane 1) and TbCDS CKO cells in absence of tetracycline for 42 h (lane 2) membranes incubated with UDP‐[^3^H]GlcNAc. O = origin, F = front. Solvent: 180:140:9:9:23 CHCl_3_ : MeOH : 30% NH_3_:1 M NH_4_Ac : H_2_O. Positions of the GPI intermediates are shown on left hand side. The data presented here are representative TLCs, of labellings done on several biological replicates along with their corresponding controls.

To investigate the possible detrimental effects on GPI anchor formation caused by limited amounts of CDP‐DAG, washed trypanosomal membranes formed from wild‐type and TbCDS CKO in the absence of tetracycline (42 h) were used in a cell‐free‐system assay with GDP‐[^3^H]Man in the presence or absence of UDP‐GlcNAc. As expected, with wild‐type membranes the presence of GDP‐[^3^H]Man allows dolichol‐phosphate‐[^3^H]Mannose (DPM) to be formed (Fig. 6A, lane 3) (Smith *et al*., 1996). The subsequent addition of UDP‐GlcNAc allows the formation of [^3^H]mannosylated GPI intermediates Man_1_GlcN‐PI (M1), Man_2_GlcN‐PI (M2), Man_3_GlcN‐PI (M3), Man_3_GlcN‐(acyl)‐PI (aM3) (Fig. 6A, lane 1). The TbCDS CKO membranes in the presence of GDP‐[^3^H]Man formed DPM (Fig. 6A, lane 4). However, in the presence of UDP‐GlcNAc, there was no M1, M2 or aM3 bands with only a very faint M3 band visible (Fig. 6A, lane 2), indicating a significant reduction in flux through the GPI pathway.

In order to clarify that this lack of GPI biosynthetic flux was due to a lack of newly synthesized PI from the ER resident PI synthase (that utilizes CDP‐DAG), a separate assay with UDP‐[^3^H]GlcNAc in the absence of GDP‐Man was conducted. As expected wild‐type membranes formed two strong bands corresponding to GlcNAc‐PI and GlcN‐PI (Fig. 6B, lane 1), indicating ample endogenous PI acceptor was available to catalyse the initial steps of the GPI pathway. In contrast the TbCDS CKO membranes showed significantly fainter bands for both GlcNAc‐PI and GlcN‐PI (Fig. 6B, lane 2), suggesting a lack of endogenous PI in the ER membranes.

Collectively, these results clearly demonstrate the reduced flux through the GPI biosynthetic pathway is caused by a shortage of PI in the ER, due to depletion of CDS activity.

### Subcellular localization of TbCDS in bloodstream form *T**. brucei*

The observed differential effects on the two major PI species caused by reduced TbCDS expression in BSF *T. brucei* suggests two separate pools of CDP‐DAG may be synthesized by distinctly localized TbCDS enzymes.

**Figure 7:**
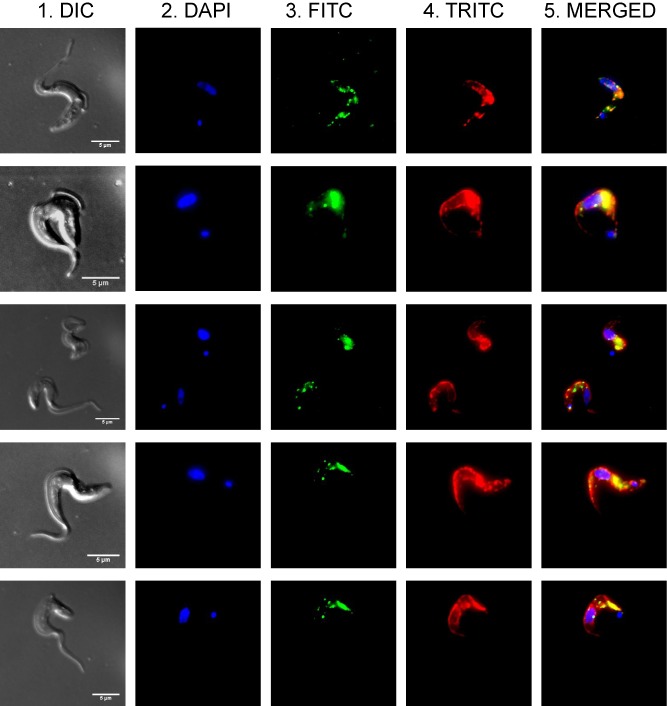
Localization of CDS‐HA in bloodstream *T**. brucei.* *T**. brucei* cells expressing CDS‐HA were co‐stained for the nuclear marker DAPI, the HA epitope and the ER marker BiP. 1. DIC image, 2. DAPI staining. 3. HA‐epitope staining and FITC detection. 4. BiP staining and TRITC detection. 5. Merged image.

To examine the subcellular localization of TbCDS, we used the TbCDS CKO cell line, as the viability conferred by the tetracycline induced copy of pLew 100 TbCDS‐HA confirms that the protein is correctly expressed and localized. The TbCDS‐HA protein was detected by Western blot of total protein (Fig. 3C, lane 2). The cells were stained against the HA‐tagged CDS and for ER localized BiP protein (Bangs *et al*., 1993; Fig. 7). The strongest signal from HA‐tagged TbCDS comes from the region between the nucleus and kinetoplast, while punctuate staining is visible throughout the cell. Since TbCDS is a membrane protein this pattern of staining indicates that it is localized to the membrane of subcellular organelles rather than the plasma membrane. Intense staining between the nucleus and kinetoplast is indicative of a Golgi localization, which has previously been shown to be the location of the downstream enzyme, PIS (Martin and Smith, 2006a) and is consistent with a requirement of CDP‐DAG for the synthesis of the bulk of PI in the cell. The perinuclear staining, and the staining throughout the cell indicate ER localization, where TbCDS could supply CDP‐DAG required for the ER localized PIS to make PI for GPI anchors.

Some co‐staining of TbCDS with the ER protein BiP does occur, particularly between the nucleus and the kinetoplast, although co‐staining is not complete and some punctuate staining of TbCDS appears to occur outwith the ER. Even within the ER, TbCDS is not evenly distributed and it may be present in specific areas of the ER (i.e. between the nucleus and the kinetoplast), possibly the mitochondrial associated membranes.

## Discussion

**Figure 8:**
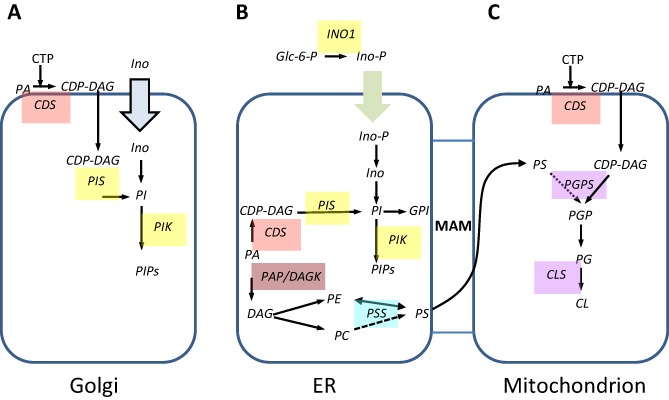
Model of revised pathways for glycerophospholipid synthesis in bloodstream form *T**. brucei.* Enzymes for which candidate genes have been identified are indicated. Block arrows indicate transporters. MAM = mitochondrial associated membranes. Metabolite abbreviations: Glc‐6‐P = glucose 6‐phosphate; PA – phosphatidic acid; CDP‐DAG – cytidine diphosphate diacylglycerol; PGP – phosphatidylglycerophosphate; PG – phosphatidylglycerol, CL – cardiolipin, PS – phosphatidylserine, PE – phosphatidylethanolamine; PI – phosphatidylinositol; PIPs – phosphorylated derivatives of phosphatidyl inositol; DAG – diacylglycerol; PE – phosphatidylethanolamine; IPC – inositol phosphorylceramide. Enzyme abbreviations: CDS – cytidine diphosphate diacylglycerol synthase, PGPS – phosphatidylglycerolphosphate synthase, CLS – cardiolipin synthase, PIS phosphatidlyinositol synthase, PIK – phosphatidylinositol kinase, PSS – phosphatidylserine synthase, PSD – phosphatidylserine decarboxylase, PAP – phosphatidic acid phosphatase, DAGK – DAG kinase. Dotted lines indicate putative pathways.

This study has given us new insight into the glycerophospholipid biosynthesis in bloodstream form *T. brucei*. Figure 8 presents a model of glycerophospholipid synthesis revised to include the alternative mechanisms indicated by our results.

CDP‐DAG synthase and PI synthase proteins are each encoded by single genes but CDP‐DAG and PI are synthesized as separate pools in the Golgi and the ER, possibly for different functions (Martin and Smith, 2006b).

A *myo*‐inositol transporter takes cytosolic inositol into the lumen of the Golgi where it is utilized to make PI via the Golgi resident PI synthase, whose active site faces the lumen of the Golgi (Gonzalez‐Salgado *et al*., 2012). The Golgi resident CDS may provide the CDP‐DAG for this PI synthase; however, the CDP‐DAG must be made on the cytosolic face of the Golgi as it uses cytosolic CTP, and therefore CDP‐DAG must be translocated across the membrane into the lumen of the Golgi.

The PI made in the Golgi is the source of bulk cellular PI, and is not used for GPI anchor synthesis, but is probably used for PIP formation by the resident PI‐4‐kinase required for post‐Golgi sorting (Martin and Smith, 2006b).

Inositol‐3‐phosphate formed from glucose 6‐phosphate by the cytosolic INO1 is taken in to the ER by an unknown transporter/mechanism, where it is dephosphorylated by inositol monophosphatase activity (Eisenberg and Parthasrathy, 1987; Martin and Smith, 2006a). This *de novo* synthesized inositol, presumably along with CDP‐DAG synthesized by a second pool of TbCDS in the ER is used by the pool of ER resident PI synthase (Martin and Smith, 2006b) In the ER, as in the Golgi, the CDP‐DAG must be translocated to the inner leaflet. The resulting PI is subsequently used for essential GPI biosynthesis (Martin and Smith, 2006a). The high flux through the PI to GPI pathway is likely be on a par with or higher than that of the PI synthesized in the Golgi, meaning that the dynamic pool of PI in the ER is very small as it is rapidly used. The ER synthesized PI is likely also used for the production of PIPs, which may occur in a subcompartment of the ER; recently described in humans (Kim *et al*., 2011).

A cell cycle stall caused by the knockout of TbCDS could indicate that PIP signalling has a role in cell cycle regulation/control in bloodstream form *T. brucei*. Additionally, depletion of GPI anchor biosynthesis as observed in this study has previously been shown to cause a cell cycle stall due to the cells being unwilling to dilute their GPI anchored VSG on the cell surface (Smith *et al*., 2009).

Previous work has indicated that the synthesis of PS via CDP‐DAG does not occur in *T. brucei* (Signorell *et al*., 2008) and in support of this, the *T. brucei* PSS enzyme shows sequence similarity to the mammalian PSS2 which utilizes PE in a head‐group exchange mechanism, rather than the yeast or prokaryotic PSS proteins which are CDP‐alcohol phosphatidyltransferases (Nikawa *et al*., 1987; Johnson *et al*., 1997). However, since knockdown of *de novo* synthesis of PE via the Kennedy pathway did not affect the amount of PS in bloodstream form *T. brucei* (Gibellini *et al*., 2009), it is possible that the *T. brucei* PSS is also capable of utilizing PC as a substrate. The production of PE through PS decarboxylation occurs in prokaryotes, yeast, and to a limited extent in mammals (reviewed in Schuiki and Daum, 2009), but this activity does not occur in bloodstream form *T. brucei*, even under conditions of depleted PE levels (Gibellini *et al*., 2009). Surprisingly, the *T. brucei* does encode a functional PS decarboxylase as demonstrated by recombinant assays and preliminary work indicates that it is essential in bloodstream form (T.K. Smith, unpublished).

For the first time, evidence is presented here that in *T. brucei* PG must be synthesized via an alternative mechanism neither directly or indirectly involving CDP‐DAG as a lipid donor. The phosphatidylglycerol‐phosphate synthase (PGPS) in procyclic *T. brucei* has been shown to be responsible for PG production, and its downregulation causes a growth defect (Serricchio and Buetikofer, 2013). However, the enzyme's substrate preference has not been experimentally confirmed. Given the increase in PG species seen in this study under limiting CDS activity it seems unlikely that CDP‐DAG is the sole substrate for PGPS. Many enzymes of the phospholipase‐D like superfamily, which includes PGPS, indicate a lack of substrate specificity (Shibuya *et al*., 1985; Nishijima *et al*., 1988; Chang *et al*., 1998; Tan *et al*., 2012). It is therefore possible that PGPS may be able to utilize an alternative to CDP‐DAG, for example PS as the lipid donor to produce PG. PS synthesis from PE is thought to occur in the mitochondrial associated membrane of the ER (Vance, 1990; Shiao and Vance, 1995; Stone and Vance, 2000) and so it would be ideally localized to feed into the mitochondrial phospholipid pathway where both *T. brucei* PGPS and CLS are found (Serricchio and Buetikofer, 2012; 2013).

The non‐reliance upon CDG‐DAG by this branch of lipid metabolism may be a strategy by the parasites to limit CTP use, as the CTP level is already dangerously low in bloodstream form *T. brucei.*

The *T. brucei* cellular response to glycerophospholipid disruption results in excess PA or DAG, which is fed into the Kennedy Pathway and converted to PG via PS, possibly in order to maintain a balance of cellular lipid as PG is considered a relatively harmless lipid to produce in excess. Whether PS is the major source of PG and CL under normal circumstances or whether synthesis of PGP from CDP‐DAG normally produces the bulk of PG remains to be determined. In many organisms CDS is localized to the mitochondria, and there is a chance that this is also the case in bloodstream form *T. brucei*, although further colocalization experiments and subcellular fractionation would be required to confirm this.

In summary this study indicates that the CDP‐DAG synthesized is primarily utilized by PI synthases in both the ER and Golgi. This new knowledge may lead to the discovery and validation of other novel drug targets against African sleeping sickness.

## Experimental procedures

### Cloning of the *T**. brucei* TbCDS gene

A putative *CDS* gene was identified in the *T. brucei* genome database (Sanger Centre) using BlastP search with the *S. cerevisiae CDS1* genomic sequence as the query. The open reading frame was amplified from *T. brucei* genomic DNA using the forward and reverse primers 5′‐GAGAAGCTTATGAAAACAAGTCCAGCAAG‐3′ and 5′‐TGCTTAATTAACTTTTCTGTGAGAGACCTATTAAG‐3′ containing HindIII and PacI restriction sites respectively (underlined). A band of the expected size of *c*. 1.2 kb was amplified using KOD polymerase, purified using a QIAquick PCR purification kit (Qiagen) and cloned into pCR‐Blunt II TOPO (Invitrogen).

### Bioinformatics

Phylogenetic analysis was performed using Mobyle@Pasteur platform from the Institut Pasteur ‘Projets et Développements en Bioinformatique’ Team and the Ressource Parisienne en Bioinformatique Structurale (http://mobyle.pasteur.fr). Sequences were aligned using Clustal O 1.0.2: Multiple Alignment (Sievers *et al*., 2011). Alignments were manually checked and gap regions and ambiguously aligned regions were excluded from further analysis. Phylogeny was inferred using the protein sequence parsimony method from the PHYLIP package (Phlipi 3.67 ProtPars) (Felsenstein, 1989).

### *S**accharomyces cerevisae* culture

The *Saccharomyces cerevisiae* strain heterozygous for the *cds1::KanMX* knockout was created by the *Saccharomyces* Genome Deletion Project (Winzeler *et al*., 1999) and obtained from Euroscarf collection. Basic yeast methods, growth media, and routine recombinant DNA methodology were performed as previously described (Gietz *et al*., 1992; Amberg *et al*., 2005). For general growth and maintenance of cultures *Saccharomyces cerevesiae* was either grown on liquid YPD containing 1% bacto‐yeast extract, 2% bacto‐peptone and 2% dextrose, or YPD plates containing 2% bacto‐agar. Otherwise, auxotrophs were grown on complete minimal glucose medium (SC) containing 0.67% bacto‐yeast nitrogen base without amino acids (Sigma) and 2% dextrose. This was supplemented with appropriate amino acid mix to select for plasmids with auxotrophic markers. Initially the amino acids were made up as sterile stocks and added as required, but later the appropriate amino acid mixes were bought from Sigma‐Aldrich. Yeast cultures were grown at 30°C, with constant shaking when in liquid media.

### Construction of TbCDS and CDS1 pRS426‐MET25 and p405‐TEF1

TbCDS was isolated from *T. brucei* strain 427 genomic DNA using the forward and reverse primers 5′‐GAGGGATCCGATGAAAAACAAGTCCAGCAAG‐3′ and 5′‐CGGCTCGAGCCTATTCTGTGAGAGACCTATTAAGAGTCGAG‐3′ containing the HindIII and XhoI restriction sites respectively. Yeast CDS1 was isolated by colony PCR from *S. cerevisiae* using the forward and reverse primers 5′‐CCCAAGCTTATGTCTTGACAACCCTGGAGATGAA‐3′ and 5′‐CGGCTCGAGTTCAAGAGTGATTGGTCAATGATTTCTTGGTCAC‐3′ containing the HindIII and XhoI restriction sites respectively (underlined). Bands of the expected size of *c*. 1.2 kb (TbCDS) or 1.4 kb (yeast CDS1) were amplified using KOD polymerase, purified using a QIAquick PCR purification kit (Qiagen) and cloned into the yeast expression vector pRS426 MET25 (2μ URA3) or p405‐TEF1 [gift from Nicholas Buchler (Addgene plasmid #15968)] using the HindIII and XhoI restriction sites.

### Complementation of cds1::KanMX with TbCDS and yeast CDS1 by tetrad analysis and plasmid shuffling

The diploid *S. cerevisiae cds1::KanMX* heterozygous strain was transformed with pRS426‐MET25 containing either yeast *CDS1*, *T. brucei CDS*, or with the empty vector, selecting transformants on SCD–Ura medium. These transformants, together with the original heterozygous *CDS1*/*cds1::KanMX* null strain were sporulated and tetrads dissected in the absence of methionine in order to allow expression of the CDS gene on the pRS426‐MET25 plasmid. While haploid *cds1::KanMX* segregants containing yeast *CDS1*on pRS426‐MET25 could be readily isolated, this was not the case with pRS426‐MET25 carrying *T. brucei CDS*. In case this reflected poor expression from the *MET25*promoter on pRS426‐MET25, the haploid *cds1::KanMX* knockouts containing yeast CDS1 on pRS416 MET25 (*MET15 his3 leu2 ura3 cds1::KanMX* [pRS425‐MET25‐CDS1]) were used to carry out a ‘plasmid shuffle’ with *TbCDS* or yeast *CDS1* expressed instead from p405‐TEF1, which carries the strong *TEF1* promoter. The yeast *cds1::KanMX* [pRS425‐MET25‐CDS1] was transformed with p405‐TEF1‐*TbCDS*, p405‐TEF1‐*CDS1* or empty p405‐TEF1, selecting for both plasmids on SC–Leu–Met–Ura. Cells were then cultured on SC–Leu–Met (i.e. + Ura) followed by plating on SC–Leu + 5‐fluoro‐orotic acid (5‐FOA) to select against the *URA3* containing pRS426‐MET25‐CDS1, thereby testing for complementation of *cds1::KanMX* by the p405‐TEF1‐based plasmids.

### Construction of TbCDS pLew vectors

The *TbCDS1* ORF was excised from Topo using HindIII and PacI and subsequently ligated into the tetracycline inducible expression vectors pLew82 and pLew100 (Wirtz *et al*., 1999) via the HindIII and PacI restriction sites.

### Construction of *T**. brucei* gene replacement cassettes

The 5′ and 3′ UTRs immediately adjacent to the *TbCDS* open reading frame were amplified from *T. brucei* genomic DNA using *KOD* polymerase. The primers 5′‐ATAAGTAAGCGGCCGCTGATTAACGCACCC‐3′ and 5′‐*CGTTTAAACTTACGGACCGTC*AAGCTTCTAGACGCTTTCACTGAAAAATGC‐3′ were used for the 5′ untranslated region (UTR), amplifying the expected 516 bp product and primers 5′‐*TGACGGTCCGTAAGTTTAAAC*GGATCCTCCATGTTGGAAGGGCACG‐3′ and 5′‐AGTAAGCGGCCGCCCTTGAAAACAATGGTGTATAC‐3′ resulted in the correct 179 bp 3′ UTR product.

These amplified products were used in a knitting PCR, resulting in a 695 bp product in which the 5′ UTR was joined to the 3′ UTR via a short BamHI–HindIII linker region contained within the described primers (italics) and a NotI site (underlined) at each end. This PCR product was ligated into pGEM‐5Zf(+) (Promega) using NotI sites, and the hygromycin (HYG) or puromycin (PAC) resistance genes were ligated between the BamHI and HindIII restriction sites. Plasmid DNA was prepared using a QIAprep Miniprep Plasmid Kit (Qiagen). After digestion with NotI the DNA was precipitated with sodium acetate/ethanol and dissolved in sterile water to a final concentration of 1 μg μl^−1^.

### Cultivation and genetic modification of *T**. brucei*

Bloodstream form *T. brucei* strain 427, which had been previously modified to express both T7 polymerase and the tetracycline repressor protein (Wirtz *et al*., 1999), are referred to here as wild‐type cells for convenience. Cells were grown HMI‐9 media supplemented with G418 (2.5 μg ml^−1^) at 37°C with 5% CO_2_ as described elsewhere (Wirtz *et al*., 1999; Chang *et al*., 2002; Roper *et al*., 2002). Transformation conditions and subsequent drug selection were also described elsewhere (Wirtz *et al*., 1999; Chang *et al*., 2002; Roper *et al*., 2002).

After drug selection with phleomycin, several *TbCDS‐HA^Ti^* clones were obtained and integration of the pLew100 ectopic copy was confirmed by PCR using primers specific to the pLew100 vector (data not shown). The first TbCDS allele was deleted from the *TbCDS‐HA^Ti^* cell line using a PAC knockout cassette. *TbCDS‐HA^Ti^* ΔTbCDS*::PAC* clones were obtained by selection with puromycin. The second TbCDS allele was replaced with the hygromycin resistance gene.

For experiments requiring tetracycline‐free conditions, Tet‐system approved fetal calf serum (Clonetech) was used. When required, tetracycline was added to the media at a final concentration of 1 μg ml^−1^. For tetracycline‐free experiments, cells were washed three times in tetracycline‐free HMI‐9 and resuspended in tetracycline‐free media at 5 × 10^4^ cells ml^−1^. Cells were counted each day and were passaged only when the density was between 2 and 3 × 10^6^ cells ml^−1^ (normally every second day).

### PCR confirmation of TbCDS knockout

The genotype of the conditional mutant was verified by PCR analysis using primer combinations to amplify sections of the knockout construct and primer combinations to amplify the TbCDS in its genomic context. If both genomic copies of the gene had indeed been deleted, there should be no product with the latter. The following forward primers were used: 5′‐AAGCTTATGACCGAGTACAAGCCCACGGTGCGCC‐3′, 5′‐AAGCTTATGAAAAAGCCTGAACTCACCGCGAC‐3′ or 5′‐GAGAAGCTTATGAAAACAAGTCCAGCAAG‐3′ along with the reverse primer 5′‐AGTAAGCGGCCGCCCTTGAAAACAATGGTGTATAC‐3′.

### RT‐PCR

To confirm TbCDS transcription, RT‐PCR was performed using the 5′‐ATAAGTAAGCGGCCGCTGATTAACGCACCC‐3′ and CDS pLew R primers to amplify the TbCDS ORF. Reverse transcription PCR was performed using the SuperScript™ One‐Step RT‐PCR kit with Platinum^®^ Taq (Invitrogen) following the manufacturers recommendation. Reaction components were 2× Reaction Mix, 10 pg–1 μg template RNA, 10 μM sense primer, 10 μM anti‐sense primer, 1 μl 0.2 μM RT/Platinum^®^ Taq Mix, DEPC treated water to 50 μl.

### CDS enzyme assays

The assay used for TbCDS activity was based on those described by Sparrow and Raetz (1985) and Wu *et al*. (1995). Washed *T. brucei* cells were harvested by centrifugation, washed twice in trypanosome dilution buffer (TDB, 5 mM KCl, 80 mM NaCl, 1 mM MgSO_4_, 20 mM NaH_2_PO_4_, 2 mM NaH_2_PO_4_, 20 mM glucose, pH 7.7) and lysed with 800 μl ice cold water. Two hundred microlitres of 5× incorporation buffer (250 mM Tris‐maleate pH 6.5; 15 mM MgCl_2_; 5 mg ml^−1^ BSA, 1.25 mM DTT) was added to the lysed cells, followed by centrifugation at 1000 rpm to remove whole cells. The cell membrane pellet was washed twice in incorporation buffer and the cell pellet resuspended in 85 μl incorporation buffer followed by sonication. Resuspended membranes were added to reaction tubes to make a final assay volume of 100 μl. The standard assay reaction consisted of washed crude membrane extract in 50 mM Tris‐maleate pH 6.5; 3 mM MgCl_2_; 1 mg ml^−1^ BSA; 0.25 mM DTT; 1 mM [^3^H]CTP at 9 μCi mM^−1^; 1.5 mM phosphatidic acid, 15 mM TX‐100 in a total volume of 150 μL. The assay was incubated at 30°C for 20 min, and stopped by addition of 666 μl 1:1 (v/v) CHCl_3_:MeOH. Radiolabelled glycolipids were extracted into the resulting 10:10:3 CHCl_3_ : MeOH : H_2_O (v/v) by shaking at 4°C for at least 1 h. Assay mixtures were spun at full speed and the supernatant evaporated to dryness. Two hundred microlitres of CHCl_3_ and 200 μl of 0.1 M HCl were then added to the tubes which were vortexed and briefly spun. The chloroform layer was removed and washed twice with 0.1 M HCl followed by evaporation to dryness. The resultant radiolabelled glycolipids were analysed by silica 60‐HPTLC in solvent system 25:15:4:2 (v/v) CHCl_3_ : MeOH : CH_3_COOH : H_2_O. Dried HPTLC plates were sprayed with En^3^hance^TM^ Spray and radiolabelled glycolipids visualized by fluorography.

### *In vivo* *T**. brucei* metabolic labelling

For each metabolic labelling, 1–2 × 10^7^ mid‐log cells were centrifuged (800 *g*, 10 min), washed in glucose free minimal essential media, before being resuspended in the same media at a final concentration of 1 × 10^7^ cells ml^−1^. Cells were labelled for 1 h at 37°C with 50 μCi ml^−1^ of either [9,10‐^3^H(N)]‐Tetradecanoic acid (myristic acid) (47 Ci mmol^−1^) (Perkin Elmer); [2‐^3^H]‐inositol (20 Ci mmol^−1^) or [1,2,3‐[^3^H]]‐glycerol (20 Ci mmol^−1^) (both American Radiolabelled Chemicals) in a shaking water bath. The cells were collected by centrifugation (800 *g* for 10 min) and samples taken for analysis. Lipids were extracted using chloroform : methanol : water (10:10:3 v/v) for 1 h, the supernatant removed and the pellet re‐extracted with chloroform : methanol (2:1 v/v) for 1 h. The supernatants were pooled and dried before samples were desalted using butanol/water partitioning. Lipids were separated by HPTLC using silica 60 HPTLC plates and chloroform : methanol : water (10:10:3 v/v) as the solvent. Radiolabelled lipids were detected by fluorography at −80°C, after spraying with En^3^hance^TM^ and using Kodak XAR‐5 film with an intensifying screen.

### Lipidomic analysis

Lipids were extracted from *T. brucei* as described previously (Richmond *et al*., 2010). Briefly, mid‐log cells are collected by centrifugation (800 *g*, 10 min) washed with PBS and suspended in 100 μl PBS and transferred to a glass tube. A total of 375 μl of 1:2 (v/v) CHCl_3_:MeOH added and vortexed. The sample is agitated vigorously for a further 10–15 min. The sample is now made biphasic by the addition of 125 μl of CHCl_3_, vortex and then 125 μl of H_2_O and vortexed again and centrifuged at 1000 *g* at RT for 5 min. The lower chloroform phase is transferred to a new glass vial, dried under nitrogen and stored in the fridge.

Total lipid extracts were dissolved in 15 μl of choloroform : methanol (1:2) and 15 μl of acetonitrile : isopropanol : water (6:7:2) and analysed with a Absceix 4000 QTrap – a triple quadrupole mass spectrometer equipped with a nanoelectrospray source.

Samples were delivered using either thin‐wall nanoflow capillary tips or a Nanomate interface in direct infusion mode (∼ 125 nl min^−1^). The lipid extracts were analysed in both positive and negative ion modes using a capillary voltage of 1.25 kV. MS/MS scanning (daughter, precursor and neutral loss scans) were performed using nitrogen as the collision gas with collision energies between 35–90 V. Each spectrum encompasses at least 50 repetitive scans.

Tandem mass spectra (MS/MS) were obtained with collision energies as follows: 35–45 V, PC in positive ion mode, parent‐ion scanning of m/z 184; 35–55 V, PI in negative ion mode, parent‐ion scanning of m/z 241; 35–65 V, PE in negative ion mode, parent‐ion scanning of m/z 196; 20–35 V, PS in negative ion mode, neutral loss scanning of m/z 87; and 40–90 V, for all glycerophospholipids (including PA, PG and CL) detected by precursor scanning for m/z 153 in negative ion mode. MS/MS fragmentation/daughter ion scanning was performed with collision energies between 35–90 V. Assignment of phospholipid species is based upon a combination of survey, daughter, precursor and neutral loss scans, as well as previous assignments (Richmond *et al*., 2010). The identity of phospholipid peaks was verified using the LIPID MAPS: Nature Lipidomics Gateway (http://www.lipidmaps.org). In order to compare the peak heights relative to each other, peaks were measured by ion intensities and their relative ratios were calculated.

### Cell‐free system assay of GPI biosynthesis

Membranes of *T. brucei* wild‐type and *TbCDS* conditional null mutants grown in the presence or absence of tetracycline for 30, 36 and 42 h were isolated and prepared as described previously (without the addition of tunicamycin prior to lysis) (Masterson *et al*., 1989), snap frozen in liquid nitrogen and stored at −80°C until required. A total of 1 × 10^7^ cell equivalents were used per assay: Briefly, membranes were washed twice in 10 ml of wash buffer (50 mM NaHEPES, pH 7.4, 250 mM KCl, 5 mM MgCl_2_, 100 μM tosyl‐l‐lysine chloromethyl ketone and 1 μg ml^−1^ leupeptin). The membranes were then resuspended by sonication in 2× incorporation buffer (2 times concentrated wash buffer containing 10 mM MnCl_2_, 1 μg ml^−1^ of tunicamycin and 1 mM DTT) and added to a reaction tube containing an equal volume of GDP‐[^3^H]Mannose (0.3 μCi per 10^7^ cell equivalents), with and without 1 mM UDP‐GlcNAc, or just [^3^H] UDP‐GlcNAc (1.0 μCi per 10^7^ cell equivalents). The cell‐free system was then sonicated briefly, incubated at 30°C for 1 h and the reaction stopped by the addition of 267 μl of chloroform: methanol (1:1 v/v). Radiolabelled glycolipid products were recovered by extraction into a chloroform/methanol/water mixture (10:10:3), evaporated to dryness, partitioned between butan‐1‐ol and water (Smith *et al*., 1996), and analysed by HPTLC in solvent system 180:140:9:9:23 (chloroform: methanol: 1 M ammonium acetate: concentrated ammonia: water). Dried HPTLC plates were sprayed with En^3^Hance^TM^ (Perkin Elmer), and radiolabelled components were visualized by fluorography at −80°C using Kodak Biomax MS films with an intensifying screen.

### Inositol analysis

Mid‐log cells were collected by centrifugation (800 *g*, 10 min) washed with TDB and stored at −20°C. Lipids were extracted from these samples by the addition of 500 μl of chloroform methanol mixture (2:1 v/v) and incubated at room temperature for 1 h. The supernatant was removed and the pellet re‐extracted with chloroform : methanol : water mixture (1:2:0.8 v/v). Pooled supernatants were dried under nitrogen prior to desalting by biphasic partitioning using 2:1 butanol : water (v/v). An internal standard of D_6_ *myo*‐inositol was added to samples prior to hydrolysis by strong acid (6 M HCl, 110°C), derivitization with TMS and analysis by GC‐MS, according to the method of Ferguson (Ferguson, 1993). *myo*‐Inositol was quantified and the mean of three separate analyses were determined.

### Immunofluorescence

At least 1 × 10^6^ cells were harvested from culture by centrifugation at 800 *g* for 10 min at room temperature and washed with TDB. Cells were fixed in PBS plus 4% paraformaldehyde and processed on slides as described previously (Martin and Smith, 2006a). Anti‐BiP was a kind gift from Jay Bangs, Buffalo. After the final washes in PBS the slides were mounted in SlowFade^®^ Gold Antifade Reagent with DAPI (Invitrogen) and a coverslip was placed over the slide. Slides were allowed to cure for 24 h at room temperature before sealing the sides with nail varnish.

All immunofluoresence microscopy was performed on a DeltaVision microscope (Applied Precision Inc.) and processed using ImageJ software (Rasband, 1997–2012).

## Supplementary Material

Supporting Information
