# Supplementary material for: The essential roles of cytidine diphosphate‐diacylglycerol synthase in bloodstream form Trypanosoma brucei
Source: Mol Microbiol. 2014 Mar 28;92(3):453–70. doi: 10.1111/mmi.12553 (PMC4114554; doi:10.1111/mmi.12553)
Supplement: Supplementary file 1 — Supporting Information [file mmi-92-453-s9.pdf]

FIG S1

```
1 ATAAGTAAGCGGCCGCTGATTACCGCACCCCGATTATGGGAAGGTATCAACTTGGTGGCGAGTGTAAGAT 7

71 TGCGCAACTGCACCATCTTTATGCTGTAAAGGGTTATTGACTTAAGGGTTCTTTATGTGTGCGTAGATAG 140

141 TTGTTTTTAAATGATTTTTTTTTGTTAACGAACGTACCCTACCTAGTTTAAAAACGGCGCAATCTTTTTT 210

211 TTTTACTCCTTTATTTTTTTTTTTGTACTTCCACGTTTTCGTAAGACGCATTTCTCCTCTATTTTTCTAT 280

281 TATTATTATTATTTTTTACCCTTAACCTTGTTGGGAGCGTAGATAGCTATTTGGTTGATTTAAGACGCAT 350

351 TAGCGTTTTCCTCATATATATTTTTTATTGGGCCAGATGATCTGTTATTGTGCGCTTTGGGCACACCACC 420

421 AGTACCGTCGAGGGGAGTGCAAATCTTCTTCAACTTTTCTACACTTAACCATTCCCACTTTTTTCTGACC 490

491 TTGCATTTTTCAGTGAAAGCTTCTAGatgaaaaacaagtccagcaagcaggttcttttgccccttcaggg 560
  1                           M  K  N  K  S  S  K  Q  V  L  L  P  L  Q  G 15

561 gaacatttctccatccgctgaatcgccaaggcccggctcactgatgacaactgagggaaggactcgcaac 630
 16  N  I  S  P  S  A  E  S  P  R  P  G  S  L  M  T  T  E  G  R  T  R  N  38

631 tacaaggaccttataaataggacagtgttctcattgatcatggcgtacttctttcttggactcgtttcca 700
 39 Y  K  D  L  I  N  R  T  V  F  S  L  I  M  A  Y  F  F  L  G  L  V  S  I 62

701 ttggtgcagaagccattatttcctccttatcatcatttgtgcttgatgtttcatgaagtcagcaggat 770
 63  G  A  E  A  I  I  F  L  L  I  I  I  L  C  L  M  F  H  E  V  S  R  I  85

771 caatcaacgggagagaaagaacaagcagctgccatcagttttcataatgaaagtatggtttctttgcact 840
 86  N  Q  R  E  R  K  N  K  Q  L  P  S  V  F  I  M  K  V  W  F  L  C  T  108

841 actatgttttccatgacagcgtatagcatacgtgaccccttggtagcaacatacccaggcgctatgaggt 910
109 T  M  F  S  M  T  A  Y  S  I  R  D  P  L  V  A  T  Y  P  G  A  M  R  Y 132

911 attacaggaatgcctggatgatcgcatttggttttccactcgttgggatggtgggttttgtgctgtcgct 980
133  Y  R  N  A  W  M  I  A  F  G  F  P  L  V  G  M  V  G  F  V  L  S  L  155

981 tcgcaaggggatgtatcgctaccagttcatgcaactcgcggcgattgtaatgactctgttgtatgtcact 1050
156  R  K  G  M  Y  R  Y  Q  F  M  Q  L  A  A  I  V  M  T  L  L  Y  V  T  178

1051 gctcaagggtacgcccaaatatcaaatgtaatgcgaggaatgctttggttcgttcttccaataagttgtg 1120
179 A  Q  G  Y  A  Q  I  S  N  V  M  R  G  M  L  W  F  V  L  P  I  S  C  V 202

1121 ttataaacaatgacacctgggcatatatttttggtaaactttttgggagaacgaagttgctggccctctc 1190
203  I  N  N  D  T  W  A  Y  I  F  G  K  L  F  G  R  T  K  L  L  A  L  S  225

1191 tcccaaaaagactgtggagggcttcgttggggctttcgtttttaccatcatatggtctttttggttcgcc 1260
226  P  K  K  T  V  E  G  F  V  G  A  F  V  F  T  I  I  W  S  F  W  F  A  248

1261 ggattcctcagctattttccccacatgtactgcgctaaaactgacttccactcagcattccactgtgaga 1330
249 G  F  L  S  Y  F  P  H  M  Y  C  A  K  T  D  F  H  S  A  F  H  C  E  K 272

1331 aggatcctctatttgtaaagcgagatgtccccatgcctgcgtttgtgcaagcgttgactttcaacaggtt 1400
273  D  P  L  F  V  K  R  D  V  P  M  P  A  F  V  Q  A  L  T  F  N  R  L 295

1401 gacaaccatcaggtgcgccagggtgcagcaacatgcacttgttttgctgcgttcgcatcccttattgcg 1470
296  T  T  I  R  C  A  R  V  Q  Q  H  A  L  V  F  A  A  F  A  S  L  I  A  318

1471 ccatttggtgggtttttcgccagcggactgaaacgagcatttaaaatgaaagattttggtgacctgatac 1540
319 P  F  G  G  F  F  A  S  G  L  K  R  A  F  K  M  K  D  F  G  D  L  I  P 342

1541 ccggacacggggggaataacagaccggatggactgccaaggcatcatgggtttttcacctgggtgtactt 1610
343  G  H  G  G  I  T  D  R  M  D  C  Q  G  I  M  G  F  F  T  W  V  Y  L  365

1611 acagtcatacgtttaccgtgatgagaattgcccttcatggcatacgatcagtagttgcgcccttcaacta 1680
366  Q  S  Y  V  Y  R  D  E  N  C  P  S  W  H  T  I  S  S  C  A  L  Q  L  388

1681 cccgaggagcaacggcgttcactattgtcgactcttaataggtctctcacagaatagTCCATGTTGGAAG 1750
389 P  E  E  Q  R  R  S  L  L  S  T  L  N  R  S  L  T  E  *               406

1751 GGCACGAGTAATAAAACGGAGCACGGCGACCTGTCATGGTTAGCGTTTAGAATTGCTGCGAGCGAGCATG 1820

1821 TTACCATAGTTGTAAGAAGCTGCTACCTGTATTGGTCTTTGCCGTTATATCGTATGCATTTGTATACACC 1890

1891 ATTGTTTTCAAGGGCGGCCGCTTACT 1916
```

FIG S2A

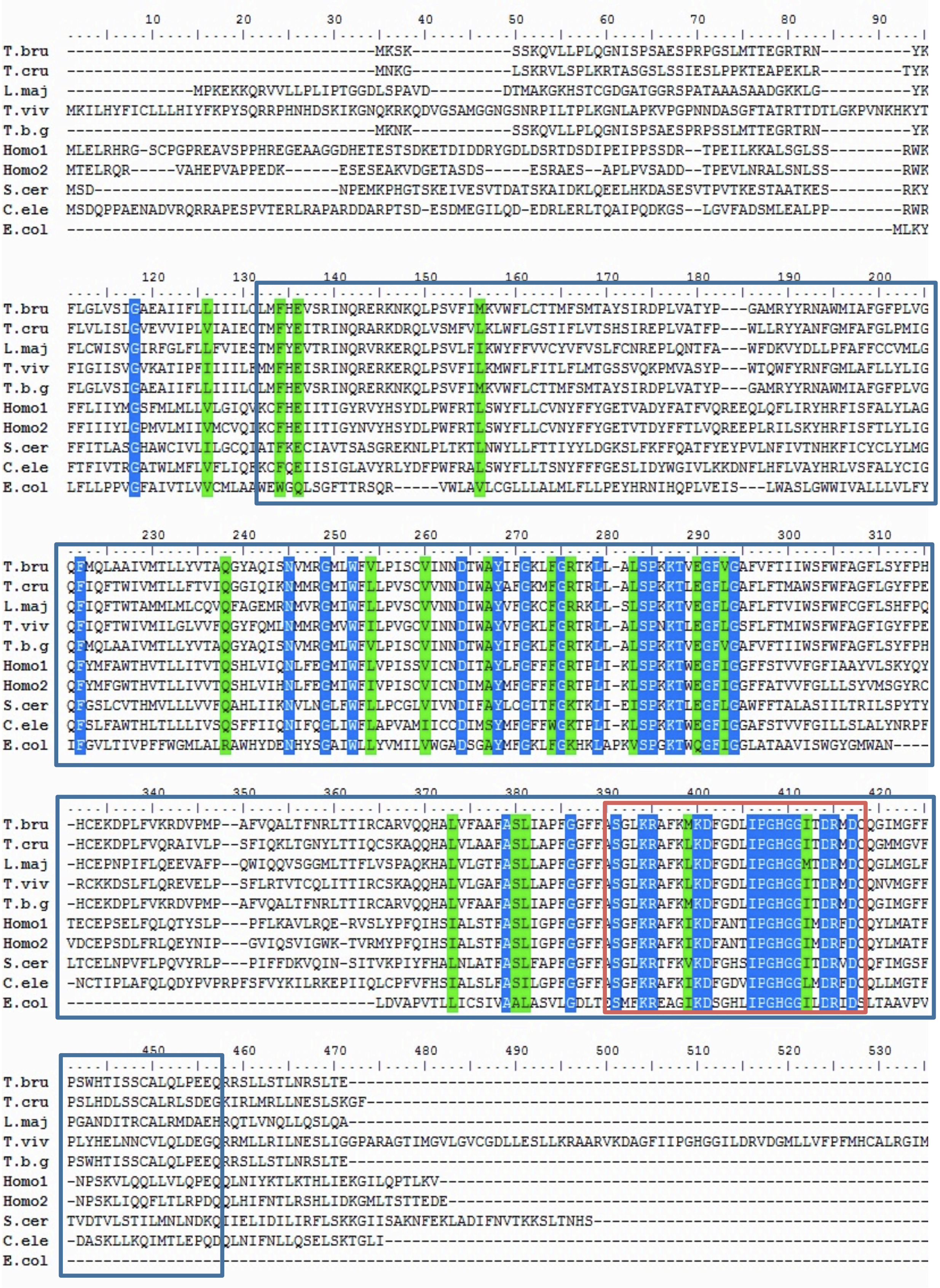
10 20 30 40 50 60 70 80 90
T.bru ----------------------------------MKSK-----------SSKQVLLPLQGNISPSAESPRPGSLMTTEGRTRN----------YK
T.cru ----------------------------------MNKG-----------LSKRVLSPLKRTASGSLSSIESLPPKTEAPEKLR---------TYK
L.maj --------------MPKEKKQRVVLLPLIPTGGDLSPAVD---------DTMAKGKHSTCGDGATGGRSPATAAASAADGKKLG----------YK
T.viv MKILHYFICLLLHIYFKPYSQRRPHNHDSKIKGNQKRKQDVGSAMGGNGSNRPILTPLKGNLAPKVPGPNNDASGFTATRTTDTLGKPVNKHKYT
T.b.g ----------------------------------MKNK-----------SSKQVLLPLQGNISPSAESPRPSSLMTTEGRTRN----------YK
Homo1 MLELRHRG-SCPGPREAVSPPHREGEAAGGDHETESTSDKETDIDDRYGDLDSRTDSDIPEIPPSSDR--TPEILKKALSGLSS--------RWK
Homo2 MTELRQR-----VAHEPVAPPEDK------ESESEAKVDGETASDS-----ESRAES--APLPVSADD--TPEVLNRALSNLSS--------RWK
S.cer MSD-------------------------NPEMKPHGTSKEIVESVTDATSKAIDKLQEELHKDASESVTPVTKESTAATKES--------RKY
C.ele MSDQPPAENADVRQRRAPESPVTERLRAPARDDARPTSD-ESDMEGILQD-EDRLERLTQAIPQDKGS--LGVFADSMLEALPP--------RWR
E.col --MLKY
120 130 140 150 160 170 180 190 200
T.bru FLGLVSIGAEAIIFLLIIILCLMFHEVSRINQRERKNKQLPSVFIMKVWFLCTTMFSMTAYSIRDPLVATYP---GAMRYYRNAWMIAFGFPLVG
T.cru FLVLISLGVEVVIPLVIAIECTMFYEITRINQRARKDRQLVSMFVLKLWFLGSTIFLVTSHSIREPLVATFP---WLLRYYANFGMFAFGLPMIG
L.maj FLCWISVGIRFGLFLLFVIESTMFYEVTRINQRVRKERQLPSVLFIKWYFFVVCYVFVSLFCNREPLQNTFA---WFDKVYDLLPFAFFCCVMLG
T.viv FIGIISVGVKATIPFIIIILRMMFHEISRINQRERKERQLPSVFILKMWFLFITLFLMTGSSVQKPMVASYP---WTQWFYRNFGMLAFLLYLIG
T.b.g FLGLVSIGAEAIIFLLIIILCLMFHEVSRINQRERKNKQLPSVFIMKVWFLCTTMFSMTAYSIRDPLVATYP---GAMRYYRNAWMIAFGFPLVG
Homo1 FFLIIYMGSFMLMLLVLGIQVKCFHEIITIGYRVYHSYDLPWFRTLSWYFLLCVNYFFYGETVADYFATFVQREEQLQFLIRYHRFISFALYLAG
Homo2 FFIIIYLGPMVLMIIVMCVQIKCFHEIITIGYNVYHSYDLPWFRTLSWYFLLCVNYFFYGETVTDYFFTLVQREEPLRILSKYHRFISFTLYLIG
S.cer FFITLASGHAWCIVLILGCQIATFKECIAVTSASGREKNLPLTKTLNWYLLFTTIYYLDGKSLFKFFQATFYEYPVLNFIVTNHKFICYCLYLMG
C.ele FTFIVTRGATWLMFLVFLIQRKCFQEIISIGLAVYRLYDFPWFRALSWYFLLTSNYFFFGESLIDYWGIVLKKDNFLHFLVAYHRLVSFALYCIG
E.col LFLLPPVGFAIVTLVVCMLAAWEWGQLSGFTTRSQR-----VWLAVLCGLLLALMLFLLPEYHRNIHQPLVEIS---LWASLGWWIVALLLVLFY
230 240 250 260 270 280 290 300 310
T.bru QFMQLAAIVMTLLYVTAQGYAQISNVMRGMLWFVLPISCVINNDTWAYIFGKLFGRTKLL-ALSPKKTVEGFVGAFVFTIIWSFWFAGFLSYFPH
T.cru QFIQFTWIVMTLLFTVIQGGIQIKNMMRGMIWFLLPVSCVVNNDIWAYAFGKMFGRTRLL-ALSPKKTLEGFLGAFLFTMAWSFWFAGFLGYFPE
L.maj QFIQFTWTAMMLMLCQVQFAGEMRNMVRGMIWFLLPVSCVVNNDIWAYVFGKCFGRRKLL-SLSPKKTVEGFLGAFLFTVIWSFWFCGFLSHFPQ
T.viv QFIQFTWIVMILGLVVFQGYFQMLNMMRGMVWFILPVGCVINNDIWAYVFGKLFGRTRLL-ALSPNKTLEGFLGSFLFTMIWSFWFAGFIGYFPE
T.b.g QFMQLAAIVMTLLYVTAQGYAQISNVMRGMLWFVLPISCVINNDTWAYIFGKLFGRTKLL-ALSPKKTVEGFVGAFVFTIIWSFWFAGFLSYFPH
Homo1 QFYMFAWTHVTLLITVTQSHLVIQNLFEGMIWFLVPISSVICNDITAYLFGFFFGRTPLI-KLSPKKTWEGFIGGFFSTVVFGFIAAYVLSKYQY
Homo2 QFYMFGWTHVTLLIVVTQSHLVIHNLFEGMIWFIVPISCVICNDIMAYMFGFFFGRTPLI-KLSPKKTWEGFIGGFFATVVFGLLLSYVMSGYRC
S.cer QFGSLCVTHMVLLLVVFQAHLIIKNVLNGLFWFLLPCGLVIVNDIFAYLCGITFGKTKLI-EISPKKTLEGFLGAWFFTALASIILTRILSPYTY
C.ele QFSLFAWTHLTLLLIVSQSFFIIQNIFQGLIWFLAPVAMIICCDIMSYMFGFFWGKTPLI-KLSPKKTWEGFIGGAFSTVVFGILLSLALYNRPF
E.col IFGVLTIVPFFWGMLALRAWHYDENHYSGAIWLLYVMILVWGADSGAYMFGKLFGKHKLAPKVSPGKTWQGFIGGLATAAVISWGYGMWAN----
340 350 360 370 380 390 400 410 420
T.bru -HCEKDPLFVKRDVPMP--AFVQALTFNRLTTIRCARVQQHALVFAAFASLIAPFGGFFASGLKRAFKMKDFGDLIPGHGGITDRMDCQGIMGFF
T.cru -HCEKDPLFVQRAIVLP--SFIQKLTGNYLTTIQCSKAQQHALVLAAFASLLAPFGGFFASGLKRAFKLKDFGDLIPGHGGITDRMDCQGMMGVF
L.maj -HCEPNPIFLQEEVAFP--QWIQQVSGGMLTTFLVSPAQKHALVLGTEASLLAPFGGFFASGLKRAFKLKDFGDLIPGHGGMTDRMDCQGLMGLF
T.viv -RCKKDSLFLQREVELP--SFLRTVTCQLITTIRCSKAQQHALVLGAFASLLAPFGGFFASGLKRAFKLKDFGDLIPGHGGITDRMDCQNVMGFF
T.b.g -HCEKDPLFVKRDVPMP--AFVQALTFNRLTTIRCARVQQHALVFAAFASLIAPFGGFFASGLKRAFKMKDFGDLIPGHGGITDRMDCQGIMGFF
Homo1 TECEPSELFQLQTYSLP---PFLKAVLRQE-RVSLYPFQIHSIALSTFASLIGPFGGFFASGFKRAFKIKDFANTIPGHGGIMDRFDCQYLMATF
Homo2 VDCEPSDLFRLQEYNIP---GVIQSVIGWK-TVRMYPFQIHSIALSTFASLIGPFGGFFASGFKRAFKIKDFANTIPGHGGIMDRFDCQYLMATF
S.cer LTCELNPVFLPQVYRLP---PIFFDKVQIN-SITVKPIYFHALNLATFASLFAPFGGFFASGLKRTFKVKDFGHSIPGHGGITDRVDCQFIMGSF
C.ele -NCTIPLAFQLQDYPVPRPFSFVYKILRKEPIIQLCPFVFHSIALSLFASILGPFGGFFASGFKRAFKIKDFGDVIPGHGGLMDRFDCQLLMGTF
E.col ---------------------------------LDVAPVTLLICSIVAALASVLGDLTESMFKREAGIKDSGHLIPGHGGILDRIDSLTAAVPV
450 460 470 480 490 500 510 520 530
T.bru PSWHTISSCALQLPEEQRRSLLSTLNRSLTE--
T.cru PSLHDLSSCALRLSDEGKIRLMRLLNESLSKGF--
L.maj PGANDITRCALRMDAEHRQTLVNQLLQSLQA--
T.viv PLYHELNNCVLQLDEGQRRMLLRILNESLIGGPARAGTIMGVLGVCGDLLESLLKRAARVKDAGFIIPGHGGILDRVDGMLLVFPFMHCALRGIM
T.b.g PSWHTISSCALQLPEEQRRSLLSTLNRSLTE--
Homo1 -NPSKVLQQLLVLQPEQQLNIYKTLKTHLIEKGILQPTLKV--
Homo2 -NPSKLIQQFLTLRPDQQLHIFNTLRSHLIDKGMLTSTTEDE--
S.cer TVDTVLSTILMNLNDKQIIELIDILIRFLSKKGIISAKNFEKLADIFNVTKKSLTNHS-------------------------------------
C.ele -DASKLLKQIMTLEPQDQLNIFNLLQSELSKTGLI--
E.col --

FIG 2B

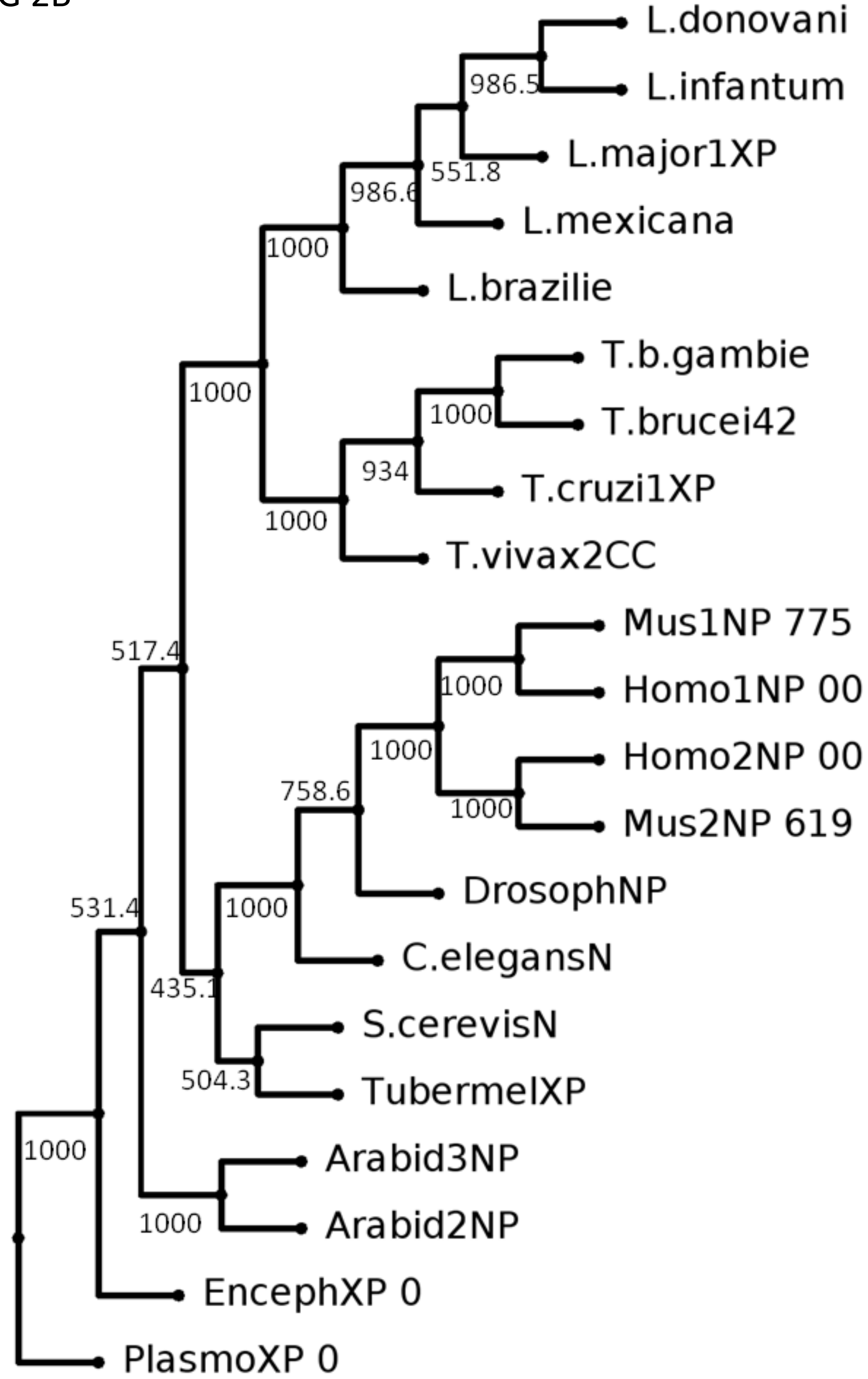
L.donovani
L.infantum
986.5
L.major1XP
551.8
986.6
L.mexicana
1000
L.brazilie
1000
T.b.gambie
T.brucei42
1000
934
T.cruzi1XP
1000
T.vivax2CC
Mus1NP 775
517.4
Homo1NP 00
1000
1000
Homo2NP 00
758.6
1000
Mus2NP 619
DrosophNP
531.4
1000
C.elegansN
435.1
S.cerevisN
504.3
TubermelXP
1000
Arabid3NP
Arabid2NP
1000
EncephXP 0
PlasmoXP 0

FIG S3A

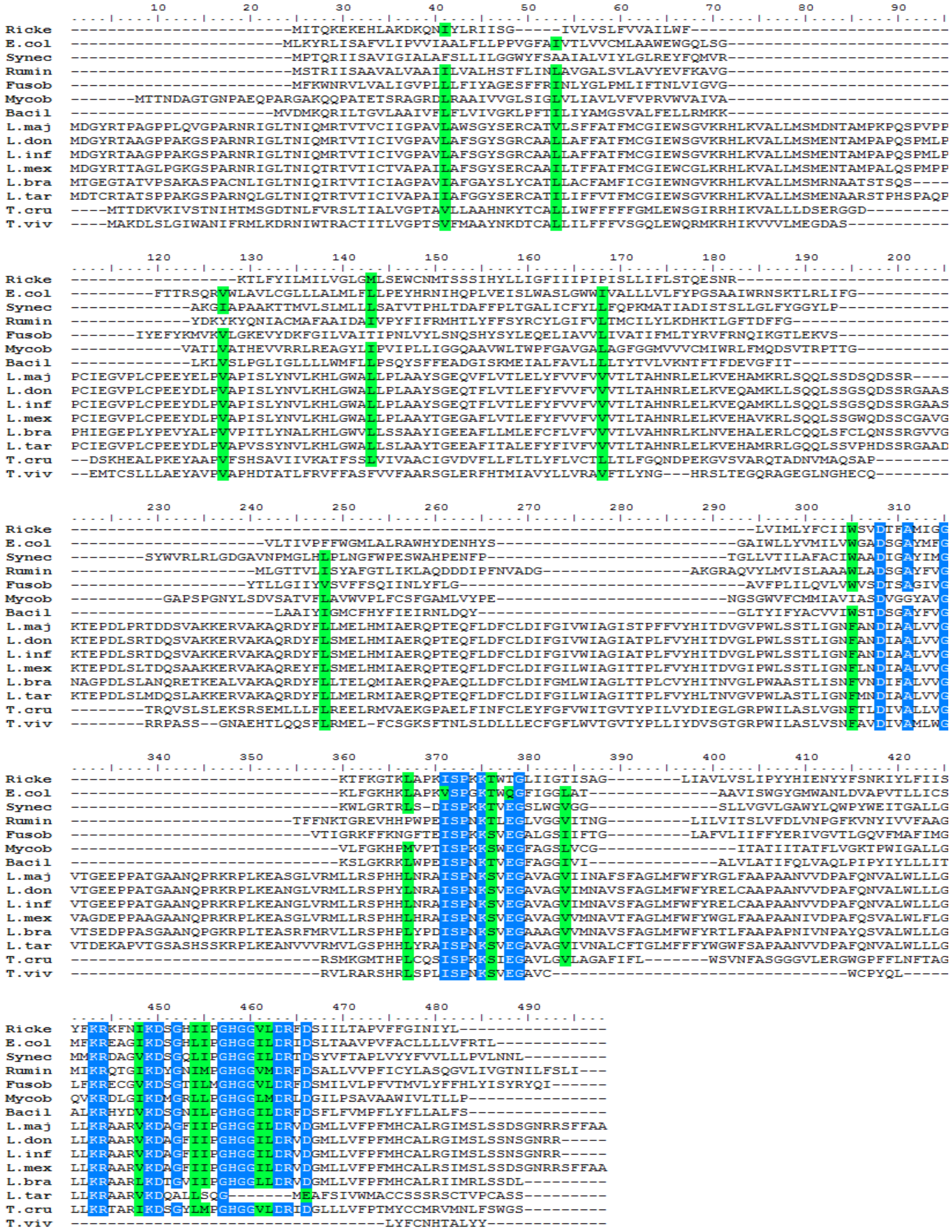
Ricke
E.col
Synec
Rumin
Fusob
Mycob
Bacil
L.maj
L.don
L.inf
L.mex
L.bra
L.tar
T.cru
T.viv

FIG S3B

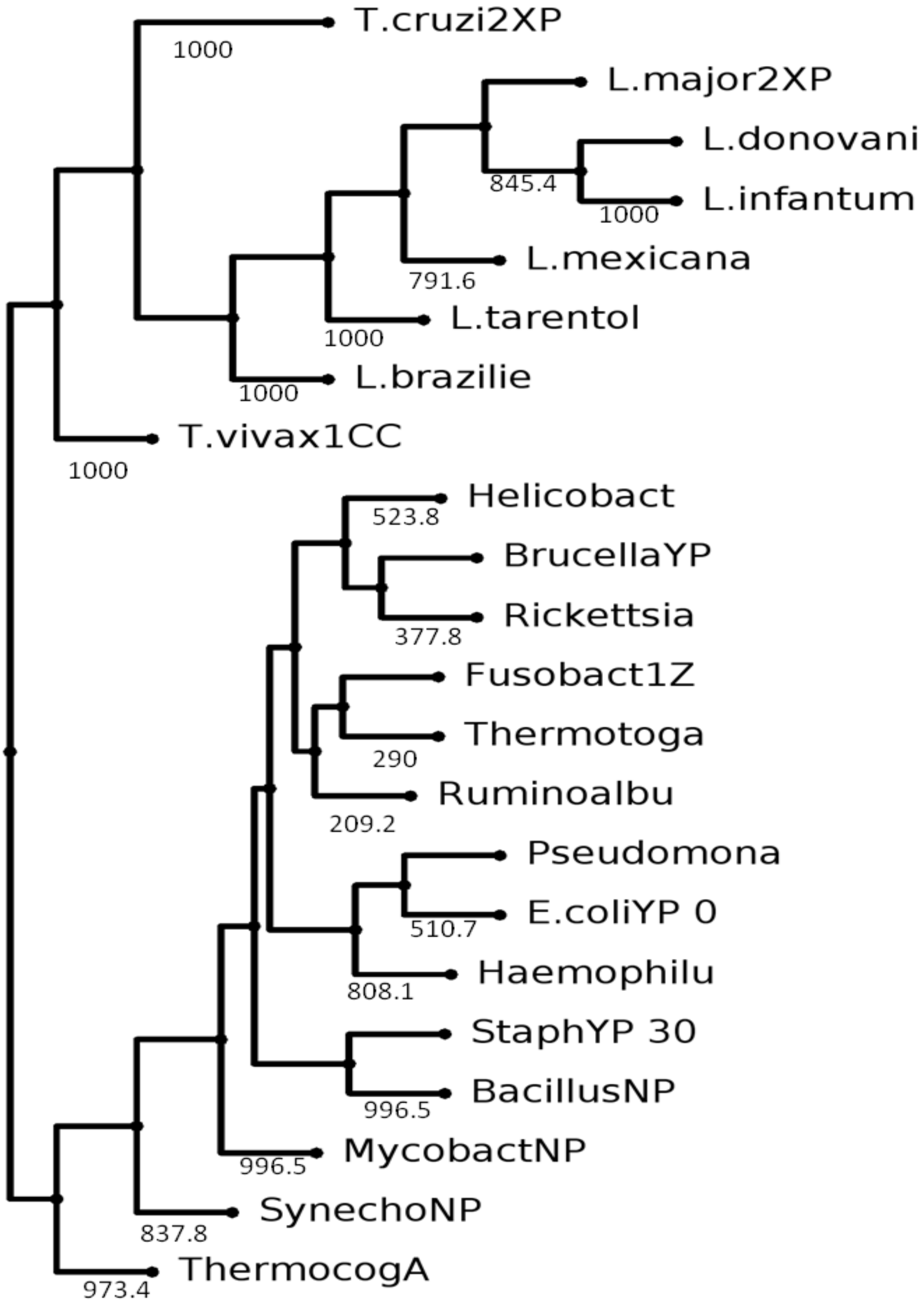

T.cruzi2XP
1000
L.major2XP
L.donovani
845.4
L.infantum
1000
L.mexicana
791.6
L.tarentol
1000
L.brazilie
1000
T.vivax1CC
1000
Helicobact
523.8
BrucellaYP
Rickettsia
377.8
Fusobact1Z
Thermotoga
290
Ruminoalbu
209.2
Pseudomona
E.coliYP 0
510.7
Haemophilu
808.1
StaphYP 30
BacillusNP
996.5
MycobactNP
996.5
SynechoNP
837.8
ThermocogA
973.4

FIG S4

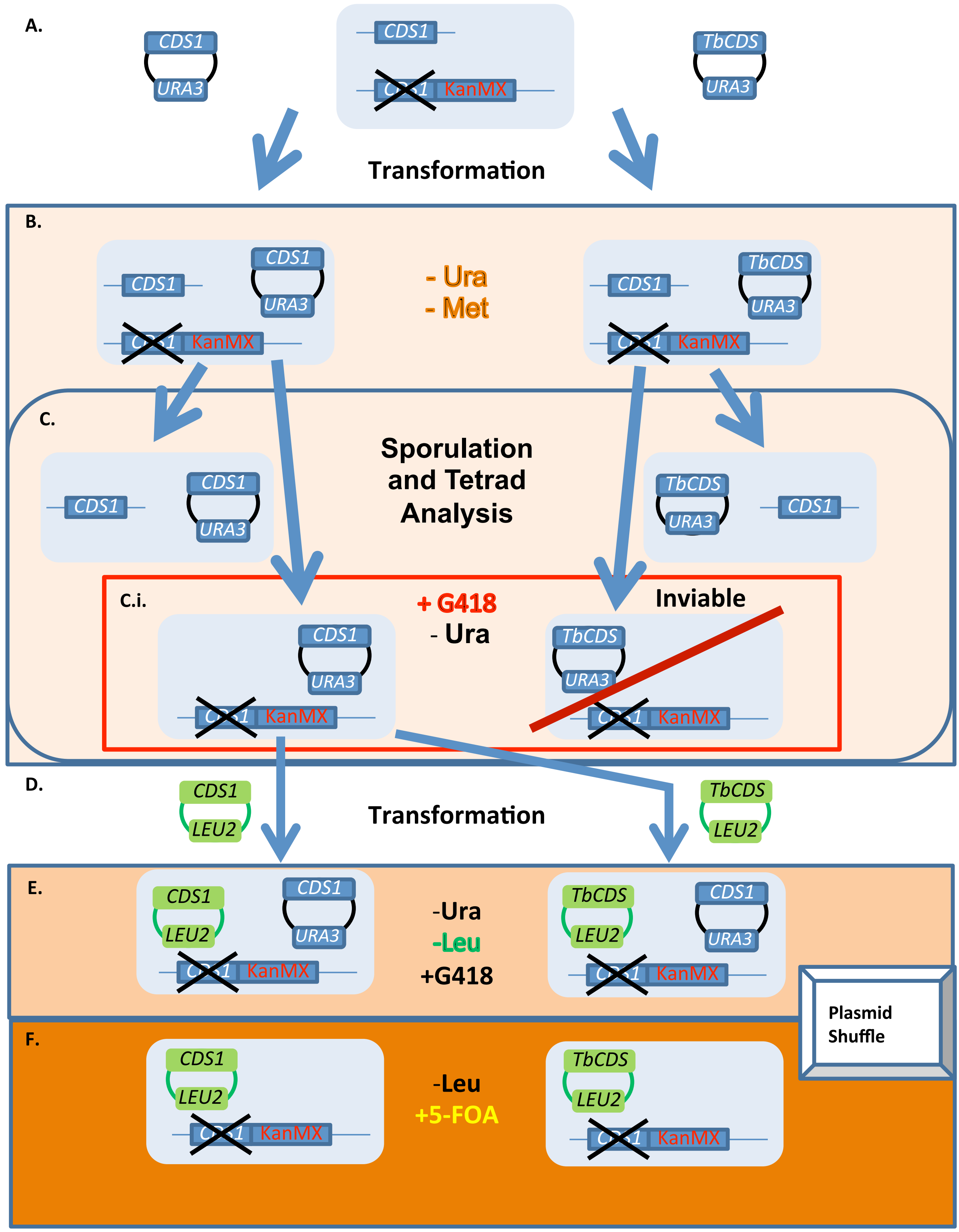

A.
CDS1
URA3
CDS1
KanMX
TbCDS
URA3
Transformation
B.
CDS1
CDS1
URA3
KanMX
- Ura
- Met
CDS1
TbCDS
URA3
KanMX
C.
Sporulation and Tetrad Analysis
CDS1
CDS1
URA3
TbCDS
URA3
CDS1
C.i.
+ G418
- Ura
Inviable
CDS1
URA3
KanMX
TbCDS
URA3
KanMX
D.
CDS1
LEU2
Transformation
TbCDS
LEU2
E.
CDS1
LEU2
CDS1
URA3
KanMX
-Ura
-Leu
+G418
TbCDS
LEU2
CDS1
URA3
KanMX
Plasmid Shuffle
F.
CDS1
LEU2
KanMX
-Leu
+5-FOA
TbCDS
LEU2
KanMX

FIG S5

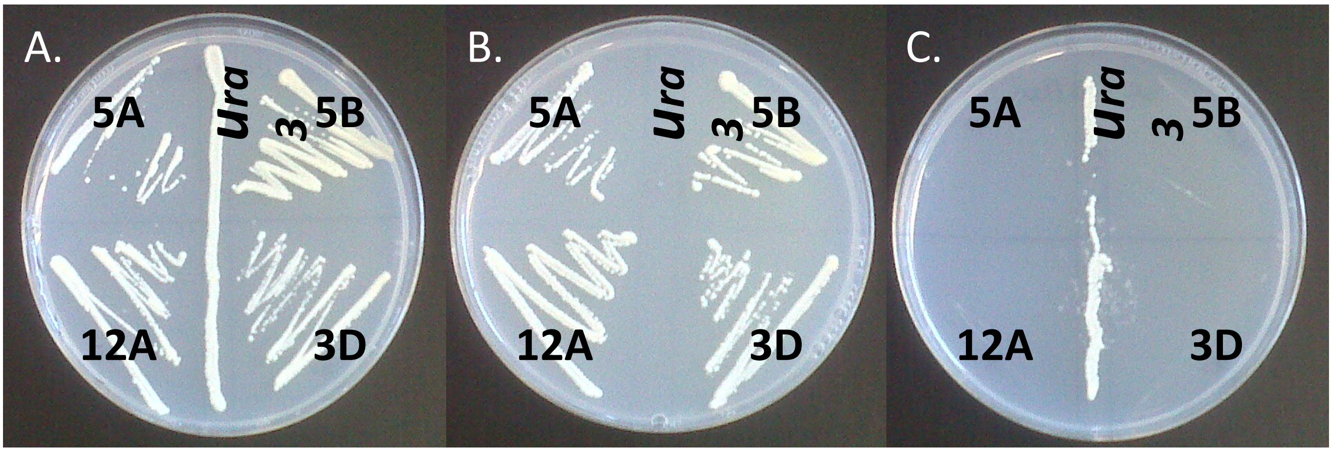

FIG S6

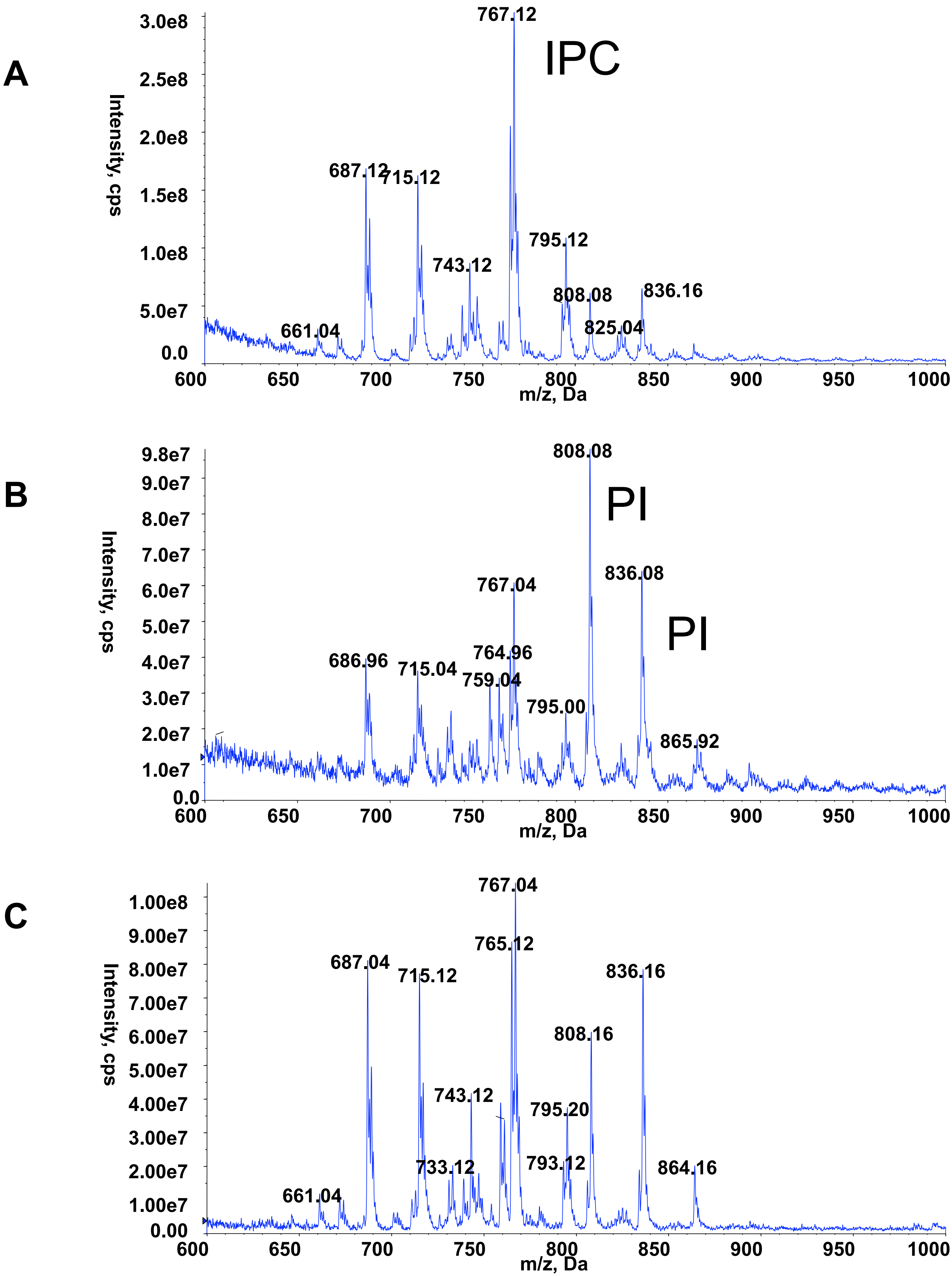

A
3.0e8
2.5e8
2.0e8
1.5e8
1.0e8
5.0e7
0.0
Intensity, cps
767.12
IPC
687.12
715.12
743.12
795.12
808.08
836.16
825.04
661.04
600
650
700
750
800
850
900
950
1000
m/z, Da
B
9.8e7
9.0e7
8.0e7
7.0e7
6.0e7
5.0e7
4.0e7
3.0e7
2.0e7
1.0e7
0.0
Intensity, cps
808.08
PI
836.08
PI
767.04
764.96
686.96
715.04
759.04
795.00
865.92
600
650
700
750
800
850
900
950
1000
m/z, Da
C
1.00e8
9.00e7
8.00e7
7.00e7
6.00e7
5.00e7
4.00e7
3.00e7
2.00e7
1.00e7
0.00
Intensity, cps
767.04
765.12
687.04
715.12
836.16
808.16
743.12
795.20
733.12
793.12
864.16
661.04
600
650
700
750
800
850
900
950
1000
m/z, Da

FIG S7

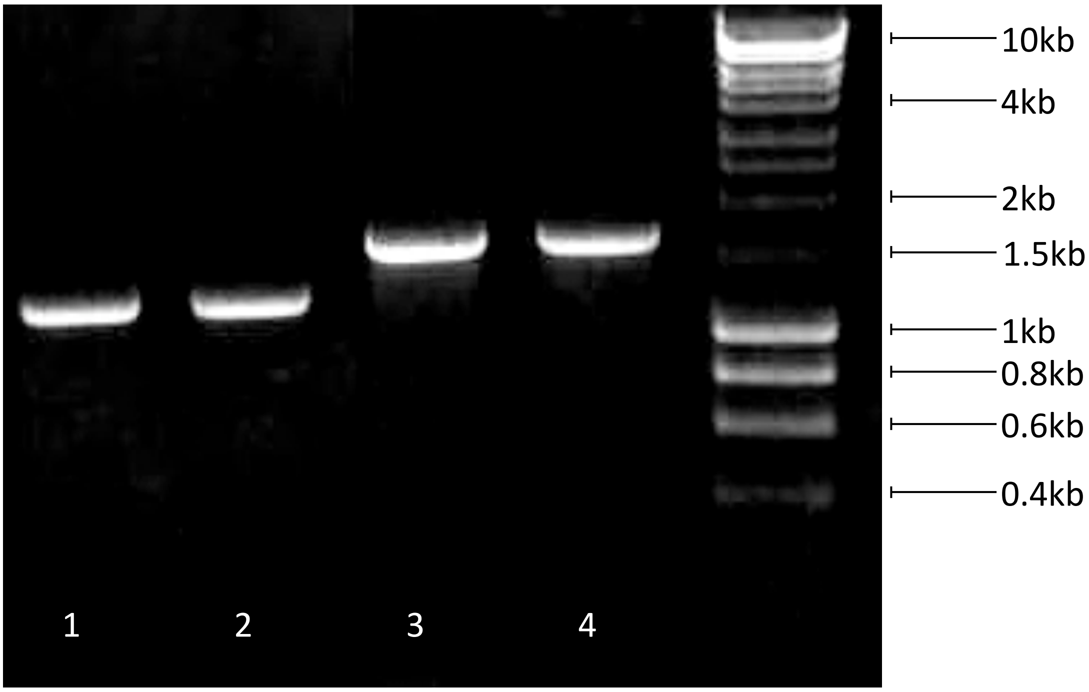

FIG S8

A

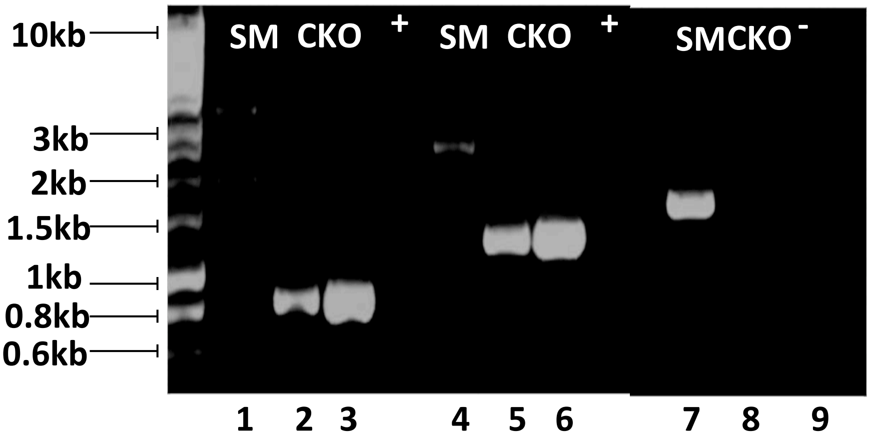
10kb
3kb
2kb
1.5kb
1kb
0.8kb
0.6kb
SM CKO +
SM CKO +
SMCKO -
1 2 3
4 5 6
7 8 9

B

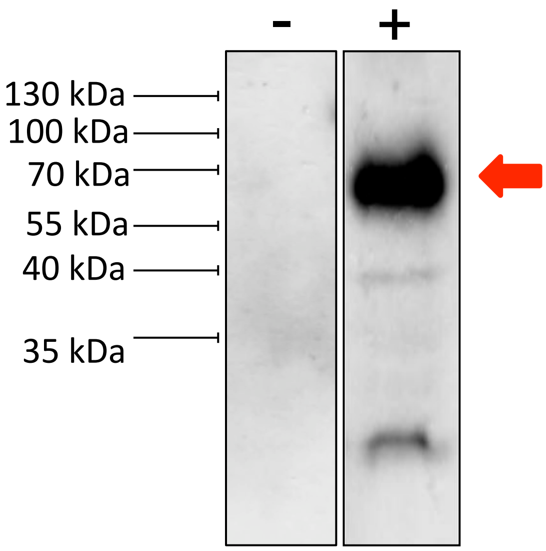
-
+
130 kDa
100 kDa
70 kDa
55 kDa
40 kDa
35 kDa

FIG S9

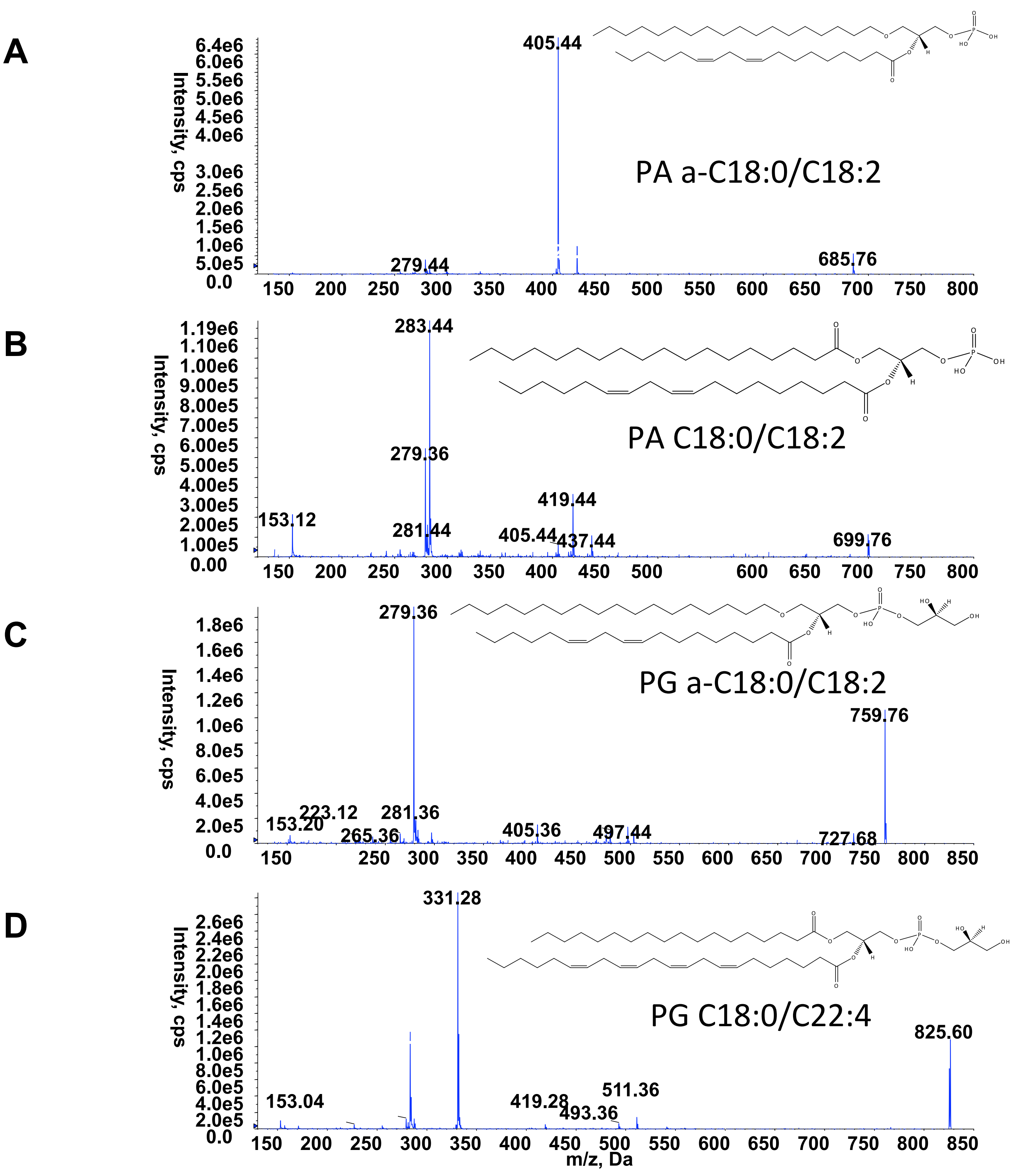

A
PA a-C18:0/C18:2
405.44
279.44
685.76
Intensity, cps
B
PA C18:0/C18:2
283.44
279.36
419.44
153.12
281.44
405.44
437.44
699.76
Intensity, cps
C
PG a-C18:0/C18:2
279.36
759.76
223.12
153.20
281.36
265.36
405.36
497.44
727.68
Intensity, cps
D
PG C18:0/C22:4
331.28
825.60
153.04
419.28
511.36
493.36
Intensity, cps
m/z, Da

FIG S10

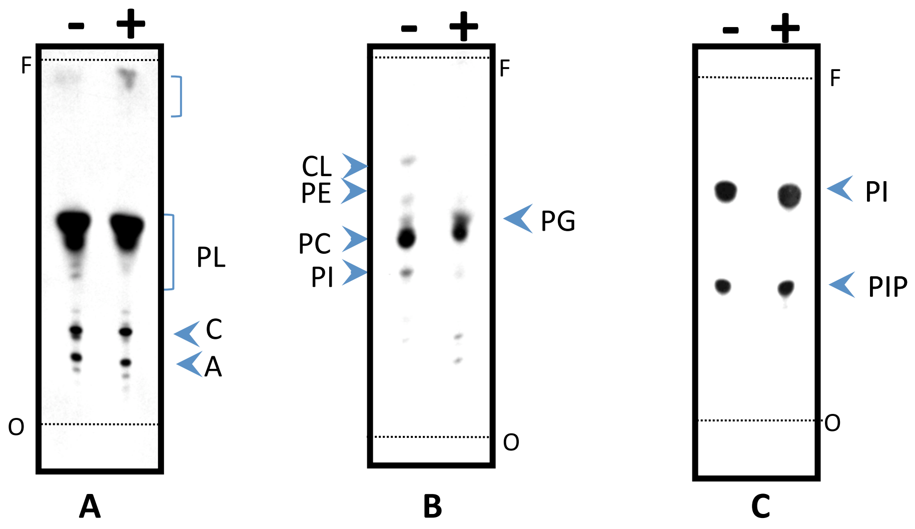

Table S1. Tetrad dissection of *MAT***a***/MATa his3Δ1/his3Δ1 leu2Δ0/leu2Δ0 lys2Δ0/LYS2 MET15/met15Δ0 ura3Δ0/ura3Δ0* [pRS425-MET25-*ScCDS1*]

	YPAD	–Met	–Ura	5-FOA	G418		YPAD	–Met	–Ura	5-FOA	G418
2A	✔	(✔)	✘	✔	✘	12A	✔	✔	✔	✘	✔
2B	✔	(✔)	✘	✔	✘	12B	✔	(✔)	✘	✔	✘
2C	–					12C	✔	(✔)	✔	✘	✔
2D	–					12D	✔	✔	✔	✔	✘
3A	–					13A	✔	(✔)	✘	✔	✘
3B	–					13B	✔	✔	✘	✔	✘
3C	✔	✔	✔	✔	✘	13C	–				
3D	✔	✔	✔	✘	✔	13D	–				
4A	–					14A	✔	✔	✘	(✘)	✘
4B	–					14B	✔	(✔)	✘	✔	✘
4C	✔	(✔)	✘	✔	✘	14C	✔	(✔)	✔	✘	✔
4D	✔	(✔)	✘	✔	✘	14D	✔	✔	✔	✘	✔
5A	✔	✔	✔	✘	✔	15A	✔	(✔)	✘	(✘)	✘
5B	✔	✔	✔	✘	✔	15B	✔	(✔)	✔	✘	✔
5C	✔	(✔)	✘	✔	✘	15C	✔	✔	✘	✔	✘
5D	✔	(✔)	✘	✔	✘	15D	✔	✔	✔	(✘)	✔
6A	–					16A	✔	✔	✔	✔	✘
6B	✔	(✔)	✘	✔	✘	16B	✔	(✔)	✘	✔	✘
6C	✔	(✔)	✔	✘	✔	16C	✔	(✔)	✔	✘	✔
6D	–					16D	–				
7A	–					17A	✔	(✔)	✘	(✔)	✘
7B	✔	(✔)	✘	✔	✘	17B	–				
7C	✔	✔	✘	✔	✘	17C	✔	(✔)	✘	✔	✘
7D	–					17D	–				
8A	–					18A	✔	✔	✘	✔	✘
8B	✔	(✔)	✔	✘	✔	18B	–				
8C	✔	✔	(✘)	✔	✘	18C	–				
8D	✔	(✔)	✔	✘	✔	18D	✔	✔	✘	✔	✘
9A	–					19A	✔	(✔)	✘	✔	✘
9B	✔	✔	✔	✘	✔	19B					
9C	✔	(✔)	✘	✔	✘	19C	✔	✔	✘	✔	✘
9D	✔	✔	(✔)	✔	✘	19D	–				
10A	✔	(✔)	✘	✔	✘	20A	–				
10B	–					20B	–				
10C	✔	✔	✔	✔	✘	20C	–	–			
10D	✔	(✔)	✔	✘	✔	20D	–				

"–" in the YPAD column indicates that the spore failed to germinate. : ✔, growth; ✗, no growth; (✔), weak growth; (✗), inconclusive. Yellow highlights indicate G418-resistant spores (i.e. have *cds1::KanMX*) that grew on –Ura plates (have the plasmid) but failed to grow on 5-FOA (required the plasmid for viability). Green highlights indicate strains that were G418 sensitive (i.e. *CDS1* wild-type), grew on –Ura plates (have the plasmid) and grew on 5-FOA (remained viable after plasmid loss).

Table S2. Tetrad dissection of *MAT***a***/MATa his3Δ1/his3Δ1 leu2Δ0/leu2Δ0 lys2Δ0/LYS2 MET15/met15Δ0 ura3Δ0/ura3Δ0* [pRS425-MET25-*TbCDS*]

	YPAD	–Met	–Ura	5-FOA	G418		YPAD	–Met	–Ura	5-FOA	G418
2A	–					12A	–				
2B	–					12B	–				
2C	✔	(✔)	✔	✔	✘	12C	✔	✔	✘	✔	(✘)
2D	✔	(✔)	✔	✔	✘	12D	✔	(✔)	✘	✔	✘
3A	–					13A	–				
3B	✔	✔	✔	✔	✘	13B	✔	(✔)	✘	✔	✘
3C	✔	(✔)	✔	✔	✘	13C	–				
3D	–					13D	✔	✔	✘	✔	✘
4A	✔	✔	✔	✔	✘	14A	✔	✔	✔	(✔)	✘
4B	✔	(✔)	✔	✔	✘	14B	–				
4C	–					14C	✔	(✔)	✔	✔	✘
4D	✔	✔	✘	✔	✔	14D	–				
5A	✔	✔	✘	✔	✘	15A	–				
5B	–					15B	✔	(✔)	✔	✔	✘
5C	–					15C	–				
5D	–					15D	–				
6A	✔	(✔)	✘	✔	✘	16A	✔	✔	✘	✔	✔
6B	✔	✔	✘	✔	✘	16B	✔	(✔)	✔	✔	✘
6C	–					16C	–				
6D	–					16D	✔	✔	✔	✔	✘
7A	✔	(✔)	✘	✔	✘	17A	✔	(✔)	✔	✔	✘
7B	✔	(✔)	✘	✔	✘	17B	–				
7C	–					17C	✔	✔	✔	✔	✘
7D	–					17D	–				
8A	✔	(✔)	✘	✔	✘	18A	–				
8B	✔	✔	✘	✔	✘	18B	–				
8C	–					18C	✔	(✔)	✘	✔	✘
8D	–					18D	✔	✔	✘	✔	✘
9A	–					19A	✔	✔	✘	✔	✘
9B	–					19B	–				
9C	✔	✔	✔	✔	(✘)	19C	✔	(✔)	✘	✔	✘
9D	–					19D	✔	(✔)	✘	✔	✘
10A	✔	✔	(✔)	✔	✘	20A	✔	(✔)	✘	✔	✘
10B	–					20B	–				
10C	✔	(✔)	(✔)	✔	✘	20C	✔	✔	✘	✔	(✘)
10D	–					20D	–				

"–" in the YPAD column indicates that the spore failed to germinate. : ✔, growth; ✗, no growth; (✔), weak growth; (✗), inconclusive. Green highlights indicate strains that were G418 sensitive (i.e. *CDS1* wild-type), grew on –Ura plates (have the plasmid) and grew on 5-FOA (remained viable after plasmid loss).
